# The effect of diet quality on the risk of developing gestational diabetes mellitus: A systematic review and meta-analysis

**DOI:** 10.3389/fpubh.2022.1062304

**Published:** 2023-01-09

**Authors:** Xiaoxia Gao, Qingxiang Zheng, Xiumin Jiang, Xiaoqian Chen, Yanping Liao, Yuqing Pan

**Affiliations:** ^1^School of Nursing, Fujian Maternity and Child Health Hospital, Fujian Medical University, Fuzhou, China; ^2^Fujian Maternity and Child Health Hospital, College of Clinical Medicine for Obstetrics and Gynecology and Pediatrics, Fujian Medical University, Fuzhou, China

**Keywords:** diet quality, diet, gestational diabetes mellitus, pregnancy, pre-pregnancy

## Abstract

**Objective:**

To examine the effect of diet quality on the risk of gestational diabetes mellitus.

**Methods:**

This review included cohort and case-control studies reporting an association between diet quality and gestational diabetes mellitus. We searched PubMed, Cochrane Library, Web of Science, Embase, PsycINFO, CINAHL Complete, Chinese Periodical Full-text Database, China National Knowledge Infrastructure, Chinese Biomedical Literature Database, and China Wanfang Database for studies published from inception to November 18, 2022. The Newcastle-Ottawa Scale was used for quality assessment, and the overall quality of evidence was assessed using the GRADEpro GDT.

**Results:**

A total of 19 studies (15 cohort, four case-control) with 108,084 participants were included. We found that better higher diet quality before or during pregnancy reduced the risk of developing gestational diabetes mellitus, including a higher Mediterranean diet (OR: 0.51; 95% CI: 0.30–0.86), dietary approaches to stop hypertension (OR: 0.66; 95% CI: 0.44–0.97), Alternate Healthy Eating Index (OR: 0.61; 95% CI: 0.44–0.83), overall plant-based diet index (OR: 0.57; 95% CI: 0.41–0.78), and adherence to national dietary guidelines (OR: 0.39; 95% CI:0.31–0.48). However, poorer diet quality increased the risk of gestational diabetes mellitus, including a higher dietary inflammatory index (OR: 1.37; 95% CI: 1.21–1.57) and overall low-carbohydrate diets (OR: 1.41; 95% CI: 1.22–1.64). After meta-regression, subgroup, and sensitivity analyses, the results remained statistically significant.

**Conclusions:**

Before and during pregnancy, higher diet quality reduced the risk of developing gestational diabetes mellitus, whereas poorer diet quality increased this risk.

**Systematic review registration:**

https://www.crd.york.ac.uk/PROSPERO/, identifier: CRD42022372488.

## 1. Introduction

Gestational diabetes mellitus (GDM) is the most prevalent medical illness in pregnancy and is defined as glucose intolerance of varying degrees, with onset or first detection during pregnancy (1). The average prevalence of GDM ranges from 9 to 30%, and up to 31.5% in some areas (2, 3). The prevalence of GDM has been progressively increasing due to changes in lifestyle and dietary structure (4). GDM carries significant short- and long-term health concerns for both mothers and their children. Mothers are at an increased risk of adverse pregnancy outcomes, such as premature rupture of membranes, infection, preterm labor, gestational hypertension, pre-eclampsia, excess amniotic fluid, and cesarean section; in severe cases, they may suffer ketoacidosis and have a lifetime risk of type 2 diabetes mellitus (T2DM), which is up to 20 times higher than that in normal pregnant women (5, 6). The offspring may have a significantly increased risk of hypoglycemia, macrosomia, neonatal epigenetic alterations, neonatal respiratory distress syndrome, and in severe cases, they may suffer high risk of fetal death (5, 6). In addition, offspring will carry a lifetime risk of obesity, and T2DM and metabolic syndrome are more common for them (7, 8). As a result, it is crucial for health care providers to work with pregnant women to prevent the development of GDM.

GDM has many influencing factors, including race or ethnicity, family history of diabetes mellitus, age at delivery, obesity, overweight, and lack of exercise (9); dietary factors also play an important role in its development (10). Diet quality is defined as the degree of adherence to dietary patterns recommended in dietary guidelines or indicators of a varied diet (11). In contrast to single food or nutrient intake, diet quality has been demonstrated to be a reasonable and important measure of total nutritional intake in several studies (12–14), and is a promising tool for examining the relationship between overall diet and diseases (15). Therefore, high diet quality reflects the achievement of more optimal nutrient intake profiles and a lower risk of diet-related non-communicable diseases (including T2DM) (16). A higher-quality diet is an important protective factor for diabetes (17) and is negatively associated with fasting glucose and glycated hemoglobin in adults with T2DM (18). A high-quality diet during pregnancy can help to decrease the risk of pathoglycemia, hypertension, pre-eclampsia, and excessive weight gain (19, 20); poor diet quality increases the risk of preterm birth, neonatal intensive care unit admissions, small for gestational age babies, low birth weight, and congenital heart defects (21, 22). However, the role of diet quality in the risk of GDM development has not been systematically evaluated. In addition, studies have found that the quality of a woman's diet does not change significantly before or during pregnancy (23). Schwingshackl et al. (24) encourages all women of childbearing age to adopt healthier eating behaviors, even before they become pregnant. Therefore, this study aimed to systematically review the available evidence regarding the relationship between diet quality and GDM before or during pregnancy.

## 2. Methods

The study protocol was registered in PROSPERO (no.: CRD42022372488; https://www.crd.york.ac.uk/PROSPERO/).

### 2.1. Search strategy

We searched PubMed, Cochrane Library, Web of Science, Embase, PsycINFO, CINAHL Complete, Chinese Periodical Full-text Database, China National Knowledge Infrastructure, Chinese Biomedical Literature Database, and China Wanfang Database for studies published from inception to November 18, 2022. In addition, references to relevant studies and review articles were manually searched to avoid missing publications. See Supplementary Table 1 for the search strategies.

### 2.2. Selection criteria

The inclusion criteria for this study were as follows: (1) Population: women before or during pregnancy who were involved in studies related to diet quality and GDM; (2) Exposure: studies that included diet quality as the exposure of interest, such as the Mediterranean diet (MD), dietary approaches to stop hypertension (DASH), Alternate Healthy Eating Index (AHEI), or other diet quality indices or scores; diet quality indices or scores referenced were based on established national or regional dietary guidelines; (3) Outcome: GDM; (4) Study design: cohort and case-control study; (5) Other inclusion criteria: results reported as the odds ratio (OR) or risk ratio (RR) with a 95% confidence interval (CI); studies using multiple dietary assessment methods were included.

The exclusion criteria were as follows: (1) studies with no diet quality scores but only described dietary patterns, such as clusters, factors or reduced rank regression analysis; (2) studies that examined single nutrients, foods, or food groups; (3) randomized controlled trials, cross-sectional and qualitative studies; (4) studies involving animals; (5) unpublished data and gray literature, including conference abstracts, papers, and patents.

### 2.3. Study selection

After duplicate removal, titles and abstracts were screened and full-text articles were obtained for further assessment. Study selection was independently conducted by two reviewers (Gao and Zheng). Any disagreement between the reviewers was discussed with a third reviewer (Jiang), who specialized in studying women's diets during the perinatal period. The PRISMA flowchart (http://www.prisma-statement.org/PRISMAStatement/FlowDiagram.aspx) were created to detail the inclusion/exclusion process.

### 2.4. Quality assessment

Two independent reviewers assessed the quality of included studies using the Newcastle-Ottawa Scale (NOS) (25). The NOS contains nine items categorized into three dimensions, including selection, comparability, and depending on the study type, outcome (cohort studies) or exposure (case-control studies). For each item, a series of response options were provided. The top-quality studies received a maximum score of one for each item, with the exception of the comparability item that received two scores. Each study had a maximum score of nine. Studies with a score ≥7 were considered to have a low risk of bias, and studies with a score of 3–6 were considered to have a moderate risk of bias. Studies with a high risk of bias were excluded from the meta-analysis. Any disagreement between the reviewers (Gao and Zheng) was resolved by a third reviewer (Jiang).

The overall quality of evidence for the prevalence of GDM in the diet quality of included studies was assessed using GRADEpro Guideline Development Tool (GDT) software based on the principles of Grading of Recommendations, Assessment, Development, and Evaluations (GRADE) (26). To assess the overall quality of the evidence, each outcome in GRADE was evaluated under various factors, such as the risk of bias, directness of evidence, consistency and precision of results, risk of publication bias, magnitude of the effect, dose-response gradient, and influence of residual plausible confounding factors. The final overall GRADE may be high, moderate, low, or very low depending on the scoring of the GRADE factors (27). The online version of the GRADE software was accessed and utilized for GRADE analysis.

### 2.5. Data extraction

Data extracted from each study included the first author, country, study design, follow-up duration/time, sample size, participants, dietary assessment tools, diagnostic criteria, key findings, OR/RR, and adjustment variables. For studies providing multiple estimates, we used the most complex model (i.e., including the largest number of confounders). If there was any disagreement during the data extraction, two researchers (Gao and Zheng) reviewed the full text and discussed it with a third reviewer (Jiang).

### 2.6. Data synthesis

The OR was used to analyze the results of this study. We utilized the following calculation from Deeks and Altman to convert values provided as RR to OR, where *p*_*c*_ is the usual occurrence rate without treatment (i.e., event rate in the control group) (28):


$$\begin{array}{l} OR=\frac{RR\left(1-p_{c}\right)}{1-p_{c}RR} \end{array}$$

ORs were log-transformed (i.e., lnOR) for analysis. Between-study heterogeneity was examined using the *Q*-test and *I*^2^ index. When *I*^2^ ≥ 50, the random effects model was used; otherwise, the fixed effects model was used.

For the purposes of the study, we conducted a subgroup analysis of the dietary assessment tools, types of participants (pregnancy or pre-pregnancy), and study design (cohort or case-control study). If significant heterogeneity remained after the analyses, we used subgroup analysis on study quality (low or moderate risk of bias) and country (developed or developing country) to identify the source of heterogeneity. Meta-regression analysis was used to identify the impact of adjustment variables on the study results (if there were more than 10 included studies); if *P* < 0.05, subgroup analysis was used for further exploration.

Publication bias was assessed using Begg's and Egger's tests if more than 10 studies used the same diet quality assessment tool. Sensitivity analyses were performed to confirm the stability of the overall results (29). All statistical analyses were conducted using the STATA software (version 14.0).

## 3. Results

### 3.1. Study selection

**Figure 1:**
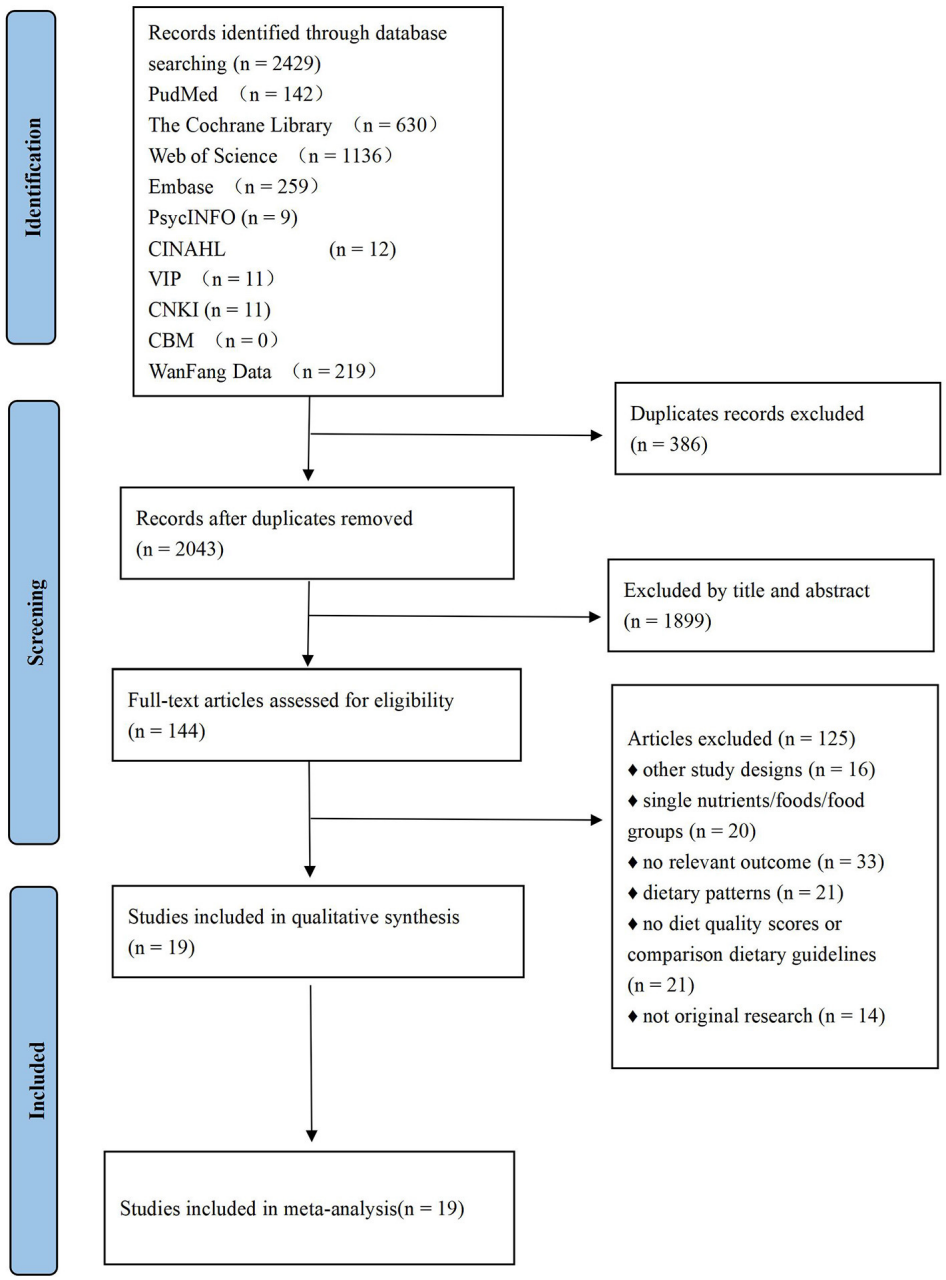
Flow diagram of included studies.

After removing duplicates, we screened the titles and abstracts of 2,429 articles for relevance. A total of 144 studies were identified as potentially eligible, and 19 were ultimately included in this systematic review. No additional articles were identified in the reference list. A flow chart is shown in Figure 1.

### 3.2. Study characteristics

The 19 included studies were published between 2012 and 2022 and included 108,084 study participants. Six studies were conducted in the United States (30–35), five in China (36–40), three in Iran (41–43), and the remaining in Japan (44), Spain (45), Iceland (46), Australia (47), and Finland (48). Six studies included pre-pregnancy (30, 31, 34, 44, 45, 47), and the remaining were pregnancy. The reported GDM diagnostic methods included a 100 g (*n* = 3) (34, 42, 45) and 75 g (*n* = 8) (36–40, 44, 46, 48) oral glucose tolerance test (OGTT), a combination of these (*n* = 5) (32, 35, 41, 43, 47), or were extrapolated from medical records (*n* = 3) (30, 31, 33).

In addition, the predominant dietary collection tool was the validated FFQ (*n* = 14). A total of eight diet quality assessment tools were included: one for Group A (i.e., the higher the diet score, the higher the diet quality) and the other for Group B (i.e., the higher the diet score, the worse the diet quality). We systematically evaluated these two groups separately. Group A included the MD, DASH, AHEI, overall plant-based diet index (overall PDI), and dietary guidelines (including China and Iceland); Group B included overall dietary inflammatory index (overall DII) and overall low-carbohydrate diets (overall LCD). Three studies simultaneously used three diet assessment tools (30, 35, 41). The characteristics and key findings of the eligible studies are presented in Table 1 and Supplementary Table 2.

**Table 1:** Eligible study characteristics.

**Study#**	**References**	**Country**	**Study design**	**Follow-up**	**Sample size**	**Participants**	**Diagnostic criteria**	**Study quality**
1	Tobias et al. (30)	United States	Cohort	1991–2002 (population-based)	15,254	Pre-pregnancy	Medical records	Moderate
2	Bao et al. (31)	United States	Cohort	1991–2001 (population-based)	21,411	Pre-pregnancy	Medical records	High
3	Izadi et al. (41)	Iran	Case-control	-	463 (cases: 200, comparison: 263)	Pregnancy	First time in the pregnancy Fasting > 95 mg/dl or 1-h > 140 mg/dl	Moderate
4	Fulay et al. (32)	United States	Cohort	2009–2013 (population-based)	1,760	Pregnancy	26–28 weeks Two-step clinical Obstetric screening	High
5	Looman et al. (47)	Australia	Cohort	2003–2015 (population-based)	3,607	Pre-pregnancy	1 h ≥ 7·8 mmol/l after a 50-g glucose load or 1 h ≥ 8·0 mmol/l after a 75-g glucose load (morning, non-fasting)	Moderate
6	Gicevic et al. (33)	United States	Cohort	1991–2001 (population-based)	21,312	Pregnancy	Medical records	High
7	Zamani et al. (43)	Iran	Case–control	-	Sample size: 460 (cases:200, comparison 260)	Pregnancy	Fasting blood glucose > 5.27 mmol/L or 1 h > 7.77 mmol/L	Moderate
8	Olmedo-Requena et al. (45)	Spain	Case-control	-	1,466 (cases: 291, comparison: 1,175)	Pre-pregnancy	100 g OGTT (24–28 weeks; fasting: 105 mg/dL; 1 h: 190 mg/dL; 2 h: 165 mg/dL; 3 h: 145 mg/dL (at least two values met or exceeded)	High
9	Shivappa et al. (42)	Iran	Case-control	-	388 (cases: 122, comparison: 266)	Pregnancy	100 g OGTT (24-28 weeks) Fasting ≥ 5.3, 1 h ≥ 10.0, 2 h ≥ 8.6 mm/l, 3 h ≥ 7.8 mm/l (at least two values met or exceeded)	Moderate
10	Li et al. (35)	United States	Cohort	1999–2002 (population-based)	1,887	Pregnancy	Fasting: 95 mg/dL, 1 h: 180 mg/dL, 2 h: 155 mg/dL, 3 h: 140 mg/dL, and/or by receipt of GDM medications (at least two values met or exceeded)	High
11	Tryggvadottir et al. (46)	Iceland	Cohort	2017–2018 (population-based)	1,015	Pregnancy	75 g OGTT (24–32 weeks) Fasting ≥ 5.1, 1 h ≥ 10.0 and 2 h ≥ 8.5 mm/l	Moderate
12	Chen et al. (36)	China	Cohort	2017–2018 (hospital-based)	1,018	Pregnancy	75 g OGTT Fasting ≥ 5.1, 1 h ≥ 10.0 or 2 h ≥ 8.5 mm/l (at least one values met or exceeded)	High
13	Dong et al. (38)	China	Cohort	From February to July 2017 (population-based)	1,455	Pregnancy	75 g OGTT (24–28 weeks) Fasting ≥ 5.1, 1 h ≥ 10.0 or 2 h ≥ 8.5 mm/l (at least one values met or exceeded)	Moderate
14	Wang et al. (39)	China	Cohort	2013–2016 (population-based)	2,099	Pregnancy	75 g OGTT (24–28 weeks) Fasting ≥ 5.1, 1 h ≥ 10.0 or 2 h ≥ 8.5 mm/l (at least one values met or exceeded)	High
15	Chen et al. (34)	United States	Cohort	1991–2001 (population-based)	20,707	Pre-pregnancy	100 g OGTT Fasting ≥ 5.0, 1 h ≥ 9.5 or 2 h ≥ 8.1 mm/l (at least two values met or exceeded)	High
16	Ding et al. (37)	China	Cohort	2013–2016 (population-based)	1,489	Pregnancy	75 g OGTT (24–28 weeks) Fasting ≥ 5.1, 1 h ≥ 10.0 or 2 h ≥ 8.5 mm/l (at least one values met or exceeded)	Moderate
17	Zhang et al. (40)	China	Cohort	2013–2016 (population-based)	2,639	Pregnancy	75 g OGTT (24–28 weeks) Fasting ≥ 5.1, 1 h ≥ 10.0 or 2 h ≥ 8.5 mm/l (at least one values met or exceeded)	High
18	Pajunen et al. (48)	Finland	Cohort	2013–2017 (hospital-based)	351	Overweight or obese pregnancy	75 g OGTT (24–28 weeks) Fasting ≥ 5.3, 1 h ≥ 10.0, 2 h ≥ 8.6 mm/l	High
19	Kyozuka et al. (44)	Japan	Cohort	2011–2014 (population-based)	9,594	Pre-pregnancy	75-g OGTT Fasting ≥ 92 mg/dL 1 h ≥ 180 mg/dL 2 h ≥ 153 mg/dL	High

OGTT, oral glucose tolerance test.

We observed that most studies adjusted for age and body mass index (BMI; 89.47 and 84.21%, respectively), and only a few studies adjusted for gestational weight gain (GWG), alcohol use, and socioeconomic status (15.79, 21.05, and 26.32%, respectively; Supplementary Figure 1).

### 3.3. Quality and GRADE assessment

**Figure 2:**
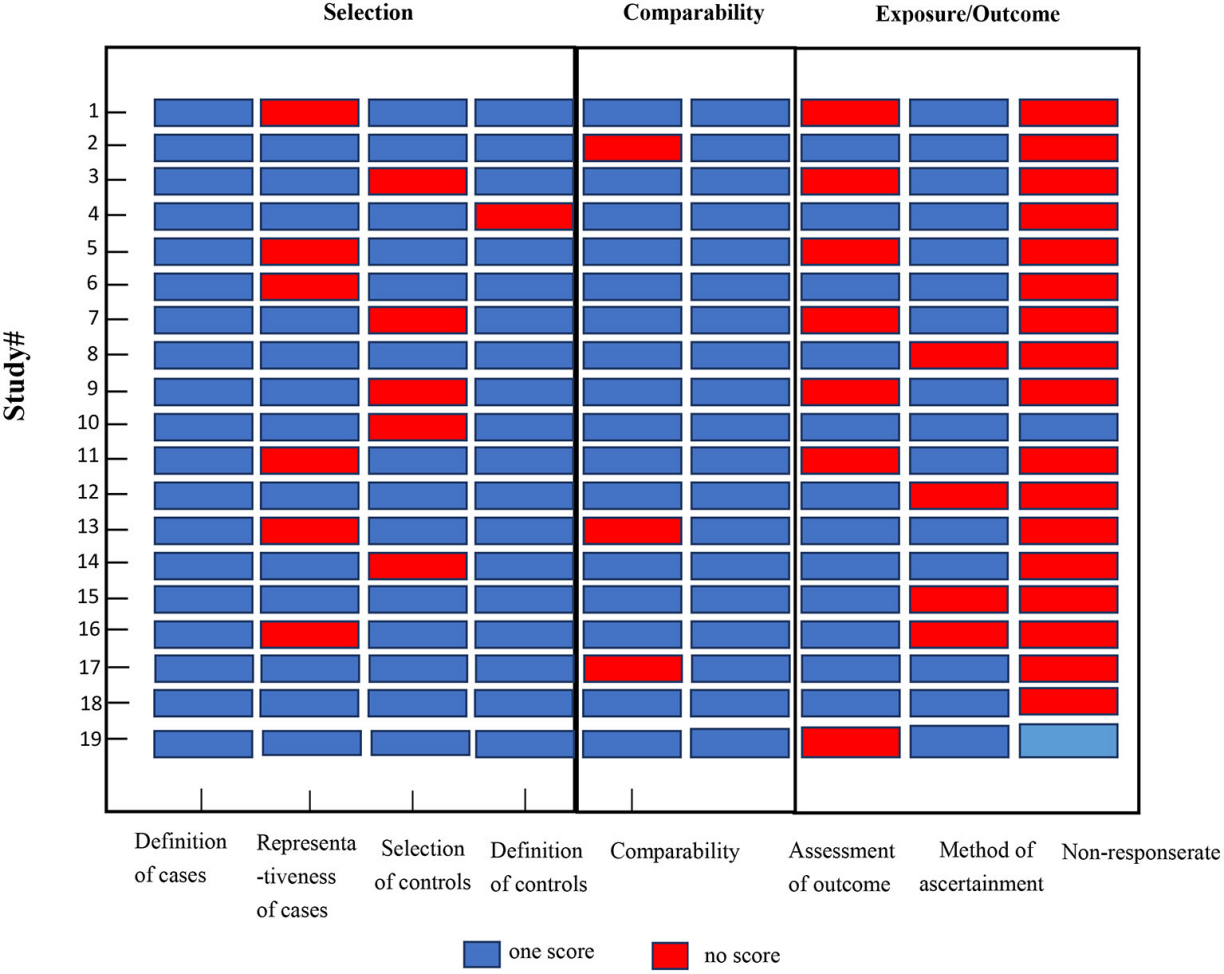
Quality assessments of the included studies. Study # is the same as in Table 1.

The eligible studies included 15 cohort studies and four case-control studies. Eleven studies had a low risk of bias [10 cohort studies (31–36, 39, 40, 44, 48) and one case-control study (45)], and eight studies had a moderate risk of bias [five cohort studies (30, 37, 38, 46, 47) and three case-control studies (41–43)] (Figure 2).

In the GRADE analysis, in Group A, the inconsistency domain was downgraded by two levels because of the presence of considerable heterogeneity between the included studies (*I*^2^ = 91.1%). We upgraded one level in the influence of residual plausible confounding factors because of the use of the most complex model. No serious issues were observed in the risk of bias, indirectness, and imprecision domains. The publication bias domain was downgraded, and there were no other additional factors. The overall GRADE recommendation in Group B (seven cohort) was “low-quality” which indicates that the true effect may be substantially different from the estimate of the effect; however, the rest were “very low-quality” which indicated that “any estimate of effect observed is very uncertain” (Table 2).

**Table 2:** GRADE assessment of the included studies.

**Good diet quality compared to poor diet quality for pregnancy or pre-pregnancy**					
**Patient or population:** pregnancy or pre-pregnancy					
**Setting:** diet quality assessment tools					
**Intervention:** good diet quality					
**Comparison:** poor diet quality					
**Outcomes**	**No. of participants (studies) follow-up**	**Certainty of the evidence (GRADE)**	**Relative effect (95% CI)**	**Anticipated absolute effects**	
				**Risk with poor diet quality**	**Risk difference with good diet quality**
**Group A**					
GDM Assessed with: diet quality assessment tools Follow-up: range 3 years to 11 years	71,244 (eight cohort)	⊕○○○^a, b, c^ Very low	**OR 0.61** (0.49–0.77)	**Moderate**	
				52 per 1,000	**20 fewer per 1,000** (26 fewer to 11 fewer)
GDM Assessed with: diet quality assessment tools	2,852 (three case-control)	⊕○○○^a, b, c^ Very low	**OR 0.37** (0.23–0.59)	396 per 1,000	**201 fewer per 1,000** (265 fewer to 117 fewer)
**Group B**					
GDM Assessed with: diet quality assessment tools Follow-up: range 5 months to 12 years	41,850 (seven cohort)	⊕⊕○○^b, c^ Low	**OR 1.38** (1.25–1.52)	68 per 1,000	**24 more per 1,000** (16 more to 32 more)
GDM Assessed with: diet quality assessment tools	388 (one case-control)	⊕○○○^c^ Very low	**OR 2.10** (1.02–4.33)	**Moderate**	
				463 per 1,000	**181 more per 1,000** (5 more to 326 more)

The risk in the exposure group (and its 95% confidence interval) is on the basis of the assumed risk in the comparison group (and its 95% CI).

CI, Confidence interval; OR, Odds ratio (i.e., Groups A and B performed subgroup analysis according to the study design).

GRADE Working Group grades of evidence. High quality: we are very confident that the true effect lies close to that of the estimate of the effect. Moderate quality: we are moderately confident in the effect estimate: the true effect is likely to be close to the estimate of the effect, but there is a possibility that it is substantially different. Low quality: our confidence in the effect estimate is limited: the true effect may be substantially different from the estimate of the effect. Very low quality: we have very little confidence in the effect estimate: the true effect is likely to be substantially different from the estimate of effect.

^a^Downgraded due to considerable heterogeneity leading to inconsistency.

^b^We upgraded one level in the influence of residual plausible confounding factors, because of using the most complex model (in the poor diet quality group, not upgraded due to only one case-control study).

^c^Downgraded due to large publication bias.

### 3.4. Meta-analysis

#### 3.4.1. Group A effects on GDM

**Figure 3:**
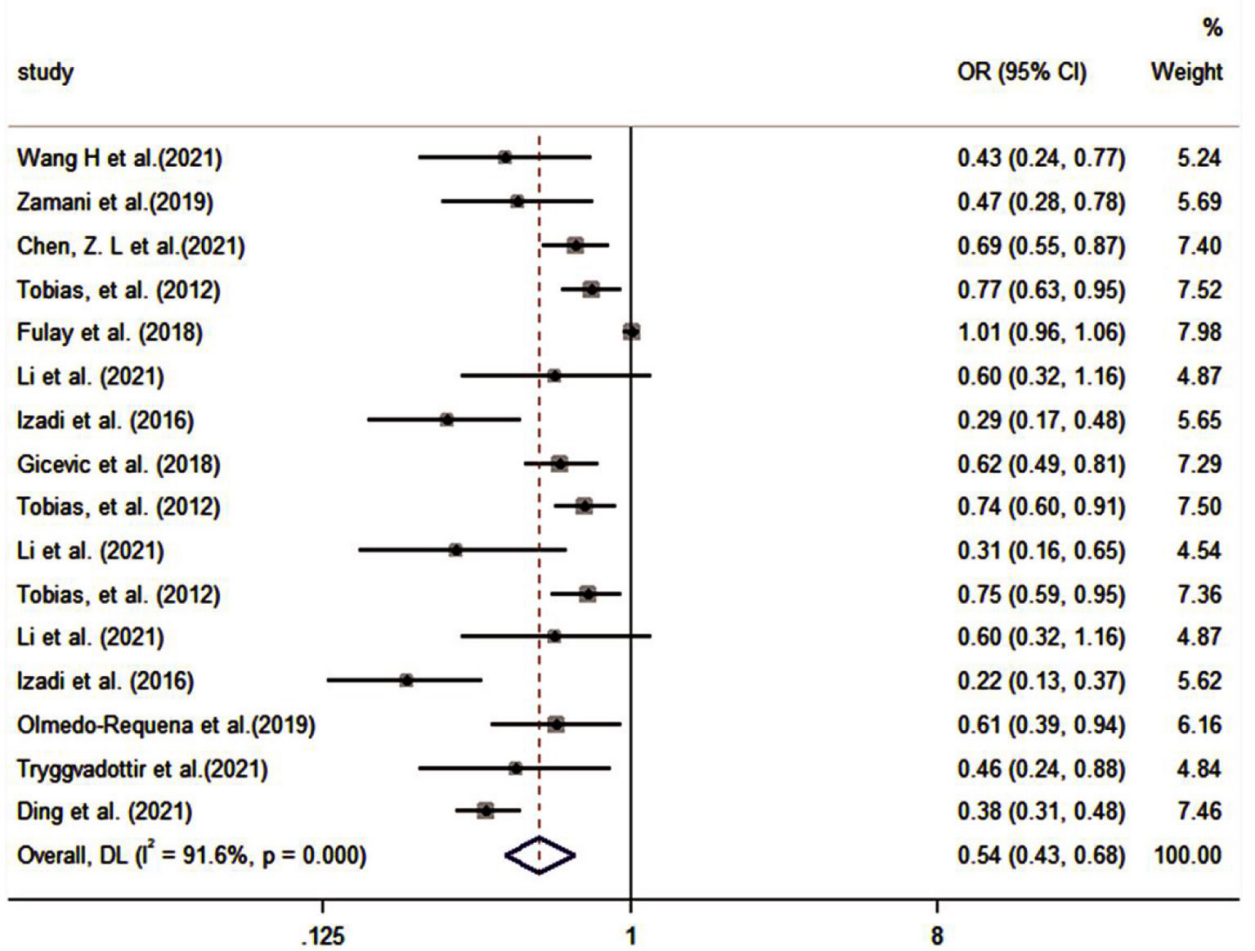
Forest plot for the association of the Group A with GDM (weights are from random-effects model).

The pooled effect size of 11 studies (30, 32–35, 37, 39, 41, 43, 45, 46) (including six assessment tools) indicated that there was a significant inverse association between high-quality diet and risk of GDM (OR: 0.54, 95% CI: 0.43–0.68, *I*^2^ = 91.6%, random effects model; Figure 3). Subgroup analysis based on dietary assessment tools indicated that the overall PDI (OR: 0.57, 95% CI: 0.41–0.78, *I*^2^ = 42.4%), DASH (OR: 0.66, 95% CI: 0.44–0.97, *I*^2^ = 90%), AHEI (OR: 0.61, 95% CI: 0.44–0.83, *I*^2^ = 65.9%), MD (OR: 0.51, 95% CI: 0.30–0.86, *I*^2^ = 82.9%), and dietary guidelines (OR: 0.39, 95% CI: 0.31–0.48, *I*^2^ = 0.0%) were all inversely associated with the risk of GDM. The results of subgroup analysis by participants showed that high-quality diet was inversely associated with GDM in both pregnancy and pre-pregnancy (OR: 0.46, 95% CI: 0.31–0.67, *I*^2^ = 93.9%; OR: 0.73, 95% CI: 0.66–0.81, *I*^2^ = 0.0%, respectively). A subgroup analysis based on the study design indicated that heterogeneity could not be eliminated (*I*^2^ = 71%). A subgroup analysis of countries and study quality was conducted to determine the main parameters involved in heterogeneity. After stratification by country, between-study heterogeneity was removed in both subgroups (*I*^2^ = 29.9%); however, heterogeneity could not be eliminated through stratification of study quality (*I*^2^ = 85.7%; Table 3).

**Table 3:** Subgroup analysis of the Group A (including dietary assessment tools, participants, country, study quality, and study design).

**Subgroup analysis of the dietary assessment tool**	**References**	**OR (95% CI)**	**Weight**
Overall PDI	Wang et al. (39)	0.43 (0.24, 0.77)	5.24
	Zamani et al. (43)	0.47 (0.28, 0.78)	5.69
	Chen et al. (34)	0.69 (0.55, 0.87)	7.40
Subgroup, DL (*I*^2^ = 42.4%, *P* = 0.176)		0.57 (0.41, 0.78)	18.34
DASH	Tobias et al. (30)	0.77 (0.63, 0.95)	7.52
	Fulay et al. (32)	1.01 (0.96, 1.06)	7.98
	Li et al. (35)	0.60 (0.32, 1.16)	4.87
	Izadi et al. (41)	0.29 (0.17, 0.48)	5.65
Subgroup, DL (*I*^2^ = 90.0%, *P* = 0.000)		0.66 (0.44, 0.97)	26.01
AHEI	Gicevic et al. (33)	0.62 (0.49, 0.81)	7.29
	Tobias et al. (30)	0.74 (0.60, 0.65)	7.50
	Li et al. (35)	0.31 (0.16, 0.65)	4.54
Subgroup, DL (*I*^2^ = 65.9%, *P* = 0.053)		0.61 (0.44, 0.83)	19.34
MD	Tobias et al. (30)	0.75 (0.59, 0.95)	7.36
	Li et al. (35)	0.60 (0.32, 1.16)	4.87
	Izadi et al. (41)	0.22 (0.13, 0.37)	5.62
	Olmedo-Requena et al. (45)	0.61 (0.39, 0.94)	6.16
Subgroup, DL (*I*^2^ = 82.9%, *P* = 0.001)		0.51 (0.30, 0.86)	24.01
Dietary guidelines	Tryggvadottir et al. (46)	0.46 (0.24, 0.88)	4.88
	Ding et al. (37)	0.38 (0.31, 0.48)	7.46
Subgroup, DL (*I*^2^ = 0.0%, *P* = 0.585)		0.39 (0.31, 0.48)	12.29
**Overall, DL (*I*^2^ = 091.6%, ***P*** = 0.000)**		**0.54 (0.43, 0.68)**	**100.0**
Pregnancy	Wang et al. (39)	0.43 (0.24, 0.77)	5.24
	Zamani et al. (43)	0.47 (0.28, 0.78)	5.69
	Fulay et al. (32)	1.01 (0.96, 1.06)	7.98
	Li et al. (35)	0.60 (0.32, 1.16)	4.87
	Izadi et al. (41)	0.29 (0.17, 0.48)	5.65
	Gicevic et al. (33)	0.62 (0.49, 0.81)	7.29
	Li et al. (35)	0.31 (0.16, 0.65)	4.54
	Li et al. (35)	0.60 (0.32, 1.16)	4.87
	Izadi et al. (41)	0.22 (0.13, 0.37)	5.62
	Tryggvadottir et al. (46)	0.46 (0.24, 0.88)	4.84
	Ding et al. (37)	0.38 (0.31, 0.48)	7.46
Subgroup, DL (*I*^2^ = 93.9%, *P* = 0.000)		0.46 (0.31, 0.67)	64.06
Pre-pregnancy	Chen et al. (34)	0.69 (0.55, 0.87)	7.40
	Tobias et al. (30)	0.77 (0.63, 0.95)	7.52
	Tobias et al. (30)	0.74 (0.60, 0.91)	7.50
	Tobias et al. (30)	0.75 (0.59, 0.95)	7.36
	Olmedo-Requena et al. (45)	0.61 (0.39, 0.94)	6.16
Subgroup, DL (*I*^2^ = 0.0%, *P* = 0.879)		0.73 (0.66, 0.81)	35.94
**Overall, DL (*I*^2^ **=** **91.6%**, ***P*** **=** 0.000)**		**0.54 (0.43, 0.66)**	**100**
Developing country	Wang et al. (39)	0.43 (0.24, 0.77)	5.24
	Zamani et al. (43)	0.47 (0.28, 0.78)	5.69
	Izadi et al. (41)	0.29 (0.17, 0.78)	5.65
	Izadi et al. (41)	0.22 (0.13, 0.37)	5.62
	Ding et al. (37)	0.38 (0.31, 0.48)	7.46
Subgroup, DL (*I*^2^ = 29.9%, *P* = 0.222)		0.35 (0.28, 0.44)	29.66
Developed country	Chen et al. (34)	0.69 (0.55, 0.87)	7.40
	Tobias et al. (30)	0.77 (0.63, 0.95)	7.52
	Fulay et al. (32)	1.01 (0.96, 1.06)	7.98
	Li et al. (35)	0.60 (0.32, 1.16)	4.87
	Gicevic et al. (33)	0.62 (0.49, 0.81)	7.29
	Tobias et al. (30)	0.74 (0.60, 0.91)	7.50
	Li et al. (35)	0.31 (0.16, 0.65)	4.54
	Tobias et al. (30)	0.75 (0.59, 0.95)	7.36
	Li et al. (35)	0.60 (0.32, 1.16)	4.87
	Olmedo-Requena et al. (45)	0.61 (0.39, 0.94)	6.16
	Tryggvadottir et al. (46)	0.46 (0.24, 0.88)	4.84
Subgroup, DL (*I*^2^ = 83.1%, *P* = 0.000)		0.68 (0.57, 0.82)	70.34
**Overall, DL (*I*^2^ **=** **91.6%**, ***P*** **=** 0.000)**		**0.54 (0.43, 0.82)**	**100.00**
Low risk of bias	Wang et al. (39)	0.43 (0.24, 0.78)	5.24
	Chen et al. (34)	0.69 (0.55, 0.87)	7.40
	Fulay et al. (32)	1.01 (0.96, 1.06)	7.98
	Li et al. (35)	0.60 (0.32, 1.16)	4.87
	Gicevic et al. (33)	0.62 (0.49, 0.81)	7.29
	Li et al. (35)	0.31 (0.16, 0.65)	4.54
	Li et al. (35)	0.60 (0.32, 1.16)	4.87
	Olmedo-Requena et al. (45)	0.61 (0.39, 0.94)	6.16
Subgroup, DL (*I*^2^ = 85.7%, *P* = 0.000)		0.62 (0.47, 0.82)	48.37
Moderate risk of bias	Zamani et al. (43)	0.47 (0.28, 0.78)	5.69
	Tobias et al. (30)	0.74 (0.60, 0.91)	7.50
	Izadi et al. (41)	0.29 (0.17, 0.78)	5.65
	Tobias et al. (30)	0.74 (0.60, 0.91)	7.50
	Tobias et al. (30)	0.75 (0.59, 0.95)	7.36
	Izadi et al. (41)	0.22 (0.13, 0.37)	5.62
	Tryggvadottir et al. (46)	0.46 (0.24, 0.88)	4.84
	Ding et al. (37)	0.38 (0.31, 0.48)	7.46
Subgroup, DL (*I*^2^ = 86.7%, *P* = 0.000)		0.49 (0.36, 0.66)	51.63
**Overall, DL (*I*^2^ **=** **91.6%**, ***P*** **=** 0.000)**		**0.54 (0.43, 0.68)**	**100.00**
Cohort study	Wang et al. (39)	0.43 (0.24, 0.78)	5.24
	Chen et al. (34)	0.69 (0.55, 0.87)	7.40
	Tobias et al. (30)	0.77 (0.63, 0.95)	7.52
	Fulay et al. (32)	1.01 (0.96, 1.06)	7.98
	Li et al. (35)	0.60 (0.32, 1.16)	4.87
	Gicevic et al. (33)	0.62 (0.49, 0.81)	7.29
	Tobias et al. (30)	0.74 (0.60, 0.91)	7.50
	Li et al. (35)	0.31 (0.16, 0.65)	4.54
	Tobias et al. (30)	0.75 (0.59, 0.95)	7.36
	Li et al. (35)	0.60 (0.32, 1.16)	4.87
	Tryggvadottir et al. (46)	0.46 (0.24, 0.88)	4.84
	Ding et al. (37)	0.38 (0.31, 0.48)	7.46
Subgroup, DL (*I*^2^ = 91.1%, *P* = 0.000)		0.61 (0.49, 0.77)	76.88
Case-control study	Zamani et al. (43)	0.47 (0.28, 0.78)	5.69
	Izadi et al. (41)	0.29 (0.17, 0.78)	5.65
	Izadi et al. (41)	0.22 (0.13, 0.37)	5.62
	Olmedo-Requena et al. (45)	0.61 (0.39, 0.94)	6.16
Subgroup, DL (*I*^2^ = 71.0%, *P* = 0.016)		0.37 (0.23, 0.59)	23.12
**Overall, DL (*I*^2^ **=** **91.6%**, ***P*** **=** 0.000)**		**0.54 (0.43, 0.68)**	**100.00**

We used meta-regression analysis for the adjustment variables (race or ethnicity, age, BMI, education, socioeconomic status, physical activity, smoking status, alcohol status, gravidity, family history of diabetes, energy intake, and GWG until the time of the study). The meta-regression analysis showed that the adjustment variables had an impact on the study results (Supplementary Table 3). Subgroup analysis of these adjustment variables indicated that physical activity, family history of diabetes, gravidity and socioeconomic status eliminated inter-group heterogeneity (0, 0.5, 8.7, and 34.1%, respectively; Supplementary Table 4).

**Figure 4:**
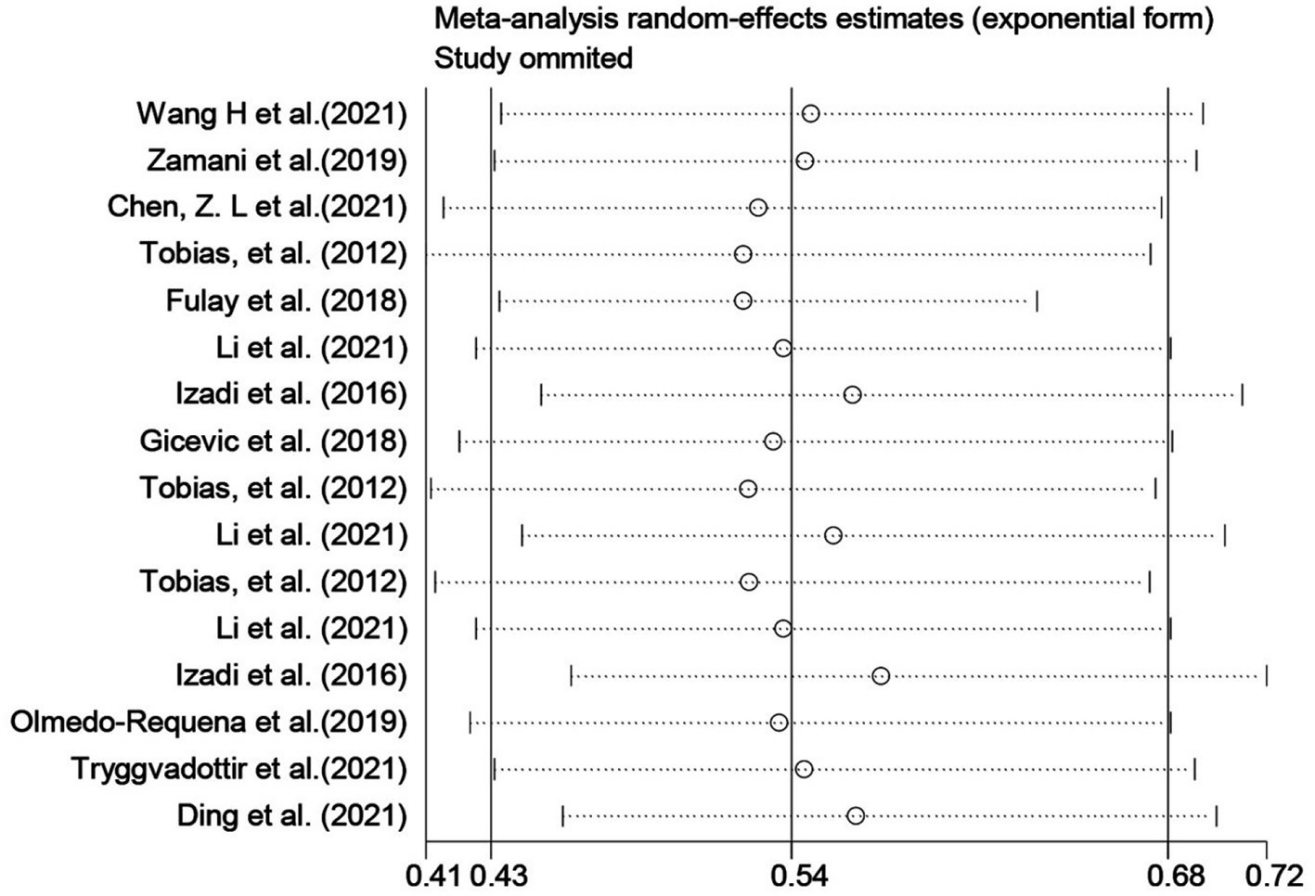
Group A sensitivity analysis.

MD and DASH were the most used diet quality assessment tools in the included studies (< 5 studies), and the significance of funnel plot asymmetry could not be tested. The results of the sensitivity analysis performed on Group A showed that the results of the systematic evaluation were reliable (Figure 4).

#### 3.4.2. Group B effects on GDM

**Figure 5:**
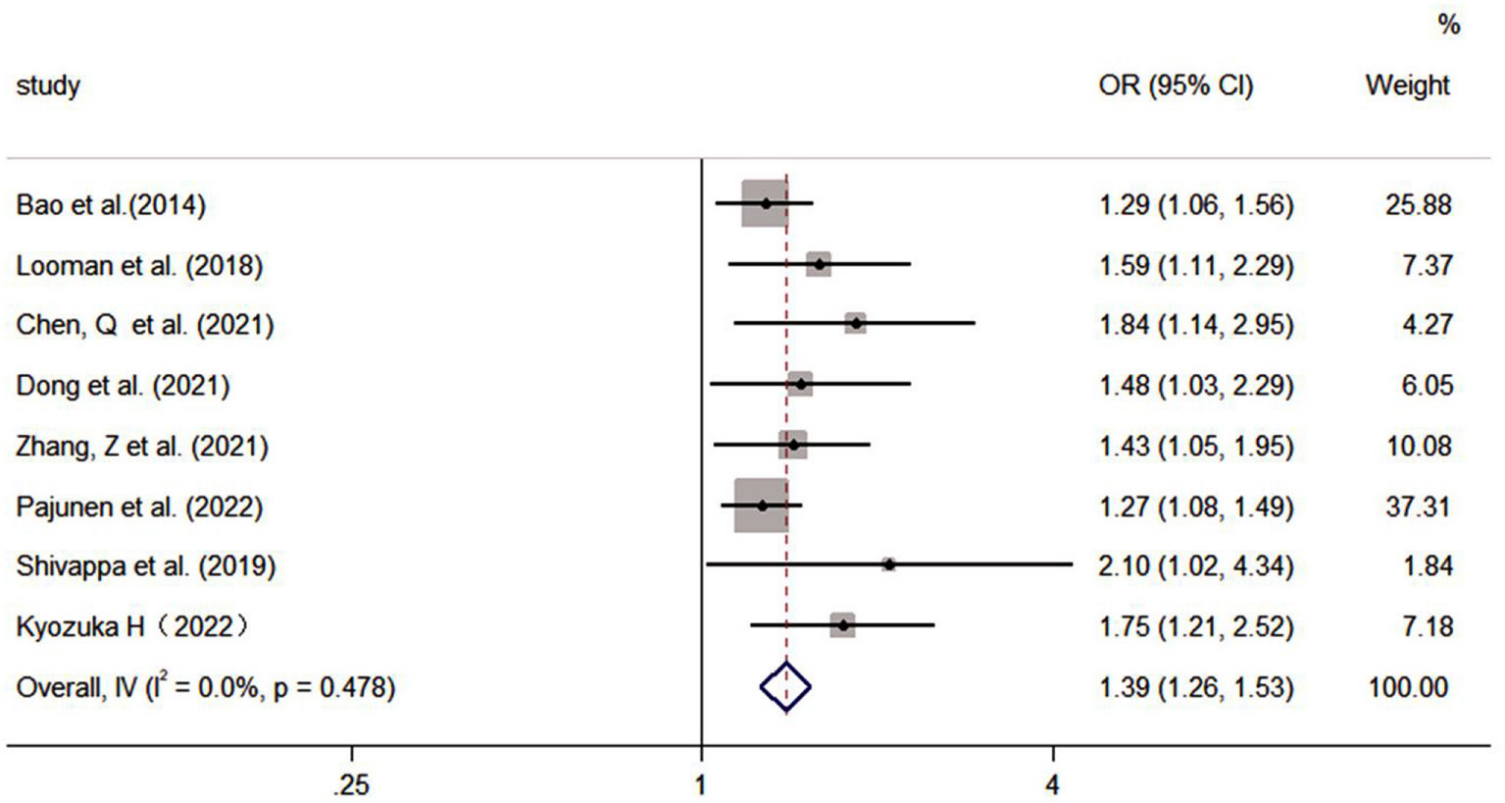
Forest plot for the association of the Group B with GDM.

The pooled effect size of eight studies (including two assessment tools) indicated that there was a significantly positive association between poor diet quality and GDM (OR: 1.39, 95% CI: 1.26–1.53, *I*^2^ = 0.0%, the fixed effects model; Figure 5). Subgroup analyses were based on dietary assessment tools, indicating that both overall LCD and DII were positively associated with GDM (OR: 1.41, 95% CI: 1.22–1.64, *I*^2^ = 0.0%; OR: 1.37, 95% CI: 1.21–1.57, *I*^2^ = 24.5%, respectively). Subgroup analyses were conducted for pregnancy and pre-pregnancy, and the results indicated that both were positively associated with GDM (OR: 1.37, 95% CI: 1.21–1.56, *I*^2^ = 0.0%; OR: 1.41, 95% CI: 1.21–1.65, *I*^2^ = 22.1%; respectively). Subgroup analysis based on study design indicated that both cohort and case-control were positively associated with GDM (OR: 1.38, 95% CI: 1.25–1.52, *I*^2^ = 0.0%; OR: 2.10; 95% CI: 1.02–4.33, *I*^2^ = 0%; respectively; Table 4).

**Table 4:** Subgroup analysis of the Group B (including dietary assessment tools, participants, and study design).

**Subgroup analysis of the dietary assessment tool**	**References**	**OR (95% CI)**	**Weight**
Overall LCD	Looman et al. (47)	1.59 (1.11, 2.29)	7.37
	Bao et al. (31)	1.29 (1.06, 1.56)	25.88
	Chen et al. (36)	1.84 (1.14, 2.95)	4.27
	Dong et al. (38)	1.48 (1.03, 2.29)	6.05
Subgroup, DL (*I*^2^ = 0.0%, *P* = 0.476)		1.41 (1.22, 1.64)	43.58
DII	Zhang et al. (40)	1.43 (1.05, 1.95)	10.08
	Pajunen et al. (48)	1.27 (1.08, 1.49)	37.31
	Shivappa et al. (42)	2.10 (1.02, 4.34)	1.84
	Kyozuka et al. (44)	1.75 (1.21, 2.52)	7.18
Subgroup, DL (*I*^2^ = 24.5%, *P* = 0.794)		1.37 (1.21, 1.57)	56.42
**Overall, DL (*I*^2^ **=** **0.0%**, ***P*** **=** 0.478)**		**1.39 (1.26, 1.53)**	**100.0**
Pregnancy	Chen et al. (36)	1.84 (1.14, 2.95)	4.27
	Dong et al. (38)	1.48 (1.03, 2.29)	6.05
	Zhang et al. (40)	1.43 (1.05, 1.95)	10.08
	Pajunen et al. (48)	1.27 (1.08, 1.49)	37.31
	Shivappa et al. (42)	2.10 (1.02, 4.34)	1.84
Subgroup, DL (*I*^2^ = 0.0%, *P* = 0.422)		1.37 (1.21, 1.56)	59.57
Pre-pregnancy	Looman et al. (47)	1.59 (1.11, 2.29)	7.37
	Bao et al. (31)	1.29 (1.06, 1.56)	25.88
	Kyozuka et al. (44)	1.75 (1.21, 2.52)	7.18
Subgroup, DL (*I*^2^ = 22.1%, *P* = 0.277)		1.41 (1.21, 1.65)	40.43
**Overall, DL (*I*^2^ **=** **0.0%**, ***P*** **=** 0.478)**		**1.39 (1.26, 1.53)**	**100**
Cohort	Chen et al. (36)	1.84 (1.14, 2.95)	4.27
	Dong et al. (38)	1.48 (1.03, 2.29)	6.05
	Zhang et al. (40)	1.43 (1.05, 1.95)	10.08
	Pajunen et al. (48)	1.27 (1.08, 1.49)	37.31
	Looman et al. (47)	1.59 (1.11, 2.29)	7.37
	Bao et al. (31)	1.29 (1.06, 1.56)	25.88
	Kyozuka et al. (44)	1.75 (1.21, 2.52)	7.18
Subgroup, DL (*I*^2^ = 0.0%, *P* = 0.510)		1.38 (1.25, 1.52)	98.16
Case-control	Shivappa et al. (42)	2.10 (1.02, 4.34)	1.84
Subgroup, DL (*I*^2^ = 0.0%)		2.10 (1.02, 4.34)	1.84
**Overall, DL (*I*^2^ = 0.0%, *P* = 0.478)**		**1.39 (1.26, 1.53)**	**100**

**Figure 6:**
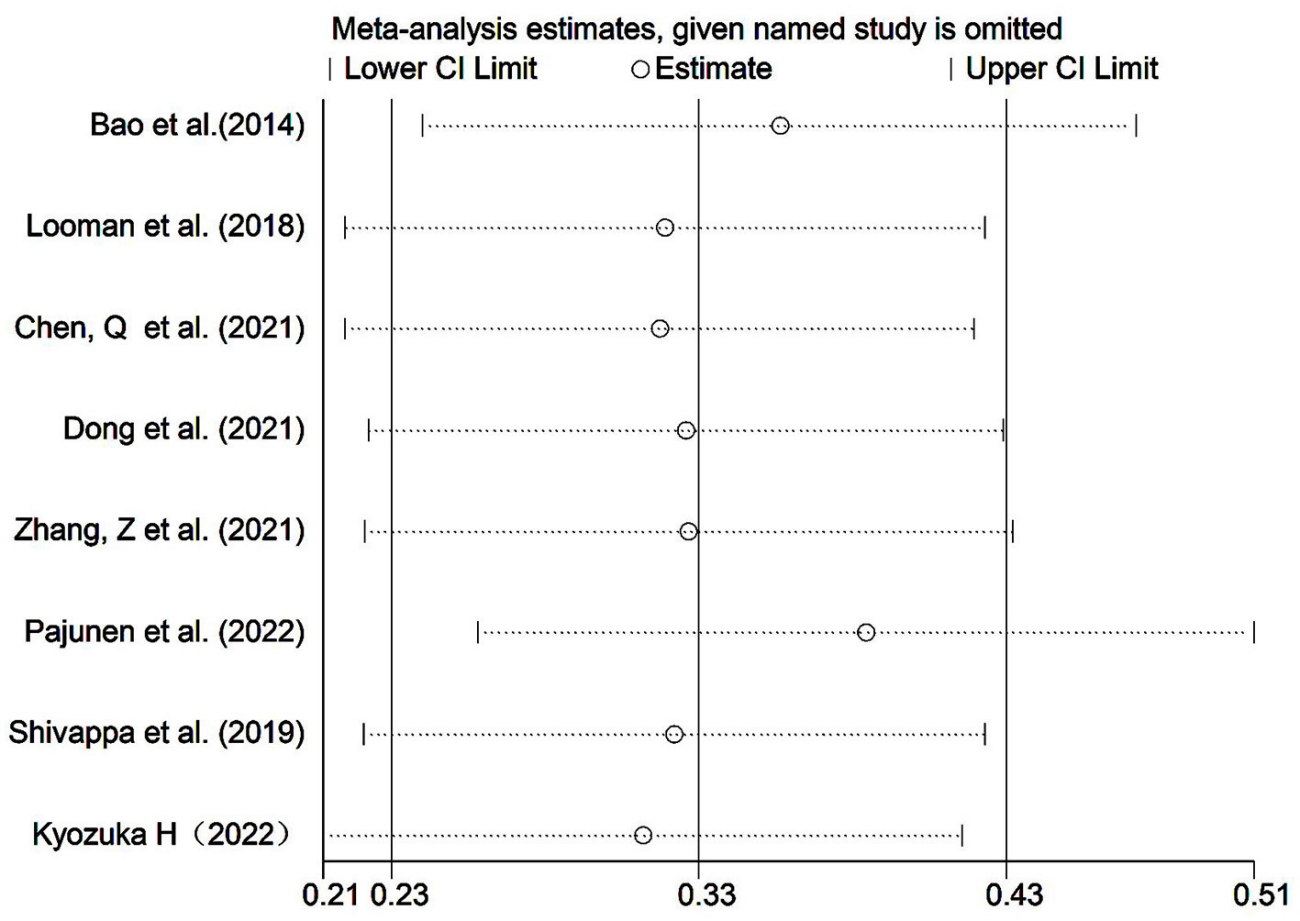
Group B sensitivity analysis.

The results of the sensitivity analysis were performed on Group B, showed that the results of the systematic evaluation were reliable (Figure 6).

## 4. Discussion

This study provides a systematic review and summary of the existing literature on the relationship between diet quality and GDM risk. The results, which included 19 studies (108,084 participants) with a total of eight diets, demonstrated that higher quality diet (MD, DASH diet, AHEI, PDI, or adherence to national dietary guidelines) before or during pregnancy reduced the risk of GDM, whereas poorer diet quality (higher DII or LCD diet) was associated with a high risk of GDM.

Although the exact molecular mechanism remains to be elucidated, the results of this study are biologically plausible. As shown in Table 5, high diet quality was mainly characterized by a higher intake of fruits, vegetables, legumes, and whole grains and a lower intake of red meat, processed meat, and trans fats. Vegetables and fruits are rich in antioxidants, fiber, polyunsaturated fatty acids, and micronutrients that can reduce glucose absorption, increase insulin secretion, and improve insulin sensitivity to assist glucose metabolism (49). Whole grain foods provide more nutrients, fiber, and phytochemicals, which serve to increase satiety, prolong the time for food to go through the digestive system, promote gut health, and reduce the glycemic response (50, 51). Vegetables, fruits, and whole grains can be directly or indirectly involved in the management of intestinal inflammation by altering intestinal flora and reducing the systemic inflammatory response (52). In contrast, red and processed meats are rich in saturated fat, hemoglobin, iron, nitrosamines, and other compounds associated with β-cell destruction, oxidative stress, insulin resistance, and GDM (53). In addition, such foods promote inflammation, alter cellular metabolic processes in the adipose tissue, liver, and pancreas, and increase the inflammatory response in GDM (54, 55).

**Table 5:** Dietary characteristics.

**Diet**	**Characterization**
**Group A (the higher the dietary score, the better the diet quality)**	
DASH diet	Based on intakes of nutrients hypothesized to alter blood pressure •Rich in fruits and vegetables •Rich in low-fat dairy food •Reduced amounts of saturated fat, total fat, and cholesterol
MD	Based on traditional eating habits in Crete, south Italy, and other Mediterranean countries•High in fruits, vegetables, cereals, and legumes •Low in saturated fats; olive oil main fat source moderate in fish •Low to moderate in dairy products •Low in red meat and meat products •Moderate in alcohol (wine)
HEI	Total fruit, whole fruit, total vegetables, dark green and orange vegetables and legumes, total grains, whole grains, dairy, meat and beans, oils, saturated fat, sodium, and empty calories
PDI	Food groups into three larger categories: •Healthy plant food groups: whole grains, fruits, vegetables, nuts, legumes, vegetable oils, and tea/coffee •Unhealthy plant food groups: fruit juices, sugar-sweetened beverages, refined grains, potatoes, and sweets/desserts •Animal food groups: animal fats, dairy, eggs, fish/seafood, meat including poultry and red/processed meat, and miscellaneous animal-based foods
Icelandic Dietary Guidelines Compliance Index for Pregnant Women	Focus on whole grains: whole-grain breads, rye breads, and other whole-grain products (such as pasta, oatmeal, barley, and whole-grain products other than bread)
Chinese Dietary Guidelines Compliance Index for Pregnant Women	Twelve components: staple food (cereals and their products, potatoes, and beans other than soybeans); vegetables; fruits; aquatic products (fish, shrimp, and shellfish); livestock and poultry meat; eggs; milk and its products; soybean and its products; nuts; vegetable cooking oil; iodized salt
**Group B (the higher the dietary score, the poorer the diet quality)**	
DII	Calculate DII scores (energy, carbohydrate, protein, total fat, fiber, cholesterol, saturated fat, mono-unsaturated fat, poly unsaturated fat, omega-3, omega-6, trans fat, niacin, thiamin, riboflavin, vitamin B12, vitamin B6, iron, magnesium, selenium, zinc, vitamin A, vitamin C, vitamin D, vitamin E, folic acid, beta carotene, garlic, turmeric, onion, and caffeine)
LCD	The percentages of fat, protein, and carbohydrate from total energy intakes: •Animal LCD score based on the proportions of energy as carbohydrate, animal protein and animal fat •Vegetable LCD score based on the percentages of energy as carbohydrate, vegetable protein and vegetable fat intakes

DASH, dietary approaches to stop hypertension; MD, Mediterranean diet; HEI, healthy Eating Index; PDI, plant-based dietindex; DII, dietary inflammatory index.

Studies have shown that dietary patterns (i.e., increased intake of higher-quality foods and reduced intake of poor-quality foods) are associated with a lower risk of GDM before and/or during pregnancy (56). Healthier eating patterns like the MD, DASH and AHEI diets can lower the risk of GDM by 15–38% (57). In contrast, a diet that is high in added sugars and organ meats, and low in fruit, vegetables, and seafood (51), a low-carbohydrate pre-pregnancy diet (58), and non-compliance with national dietary guidelines (19) were associated with a higher risk of GDM. Our findings are similar. Research on food for the prevention of GDM has received a lot of attention and has shown some promise. However, studies have shown that pre-pregnant and pregnant women may not meet the minimum dietary recommendations (59); the adherence to all food types during pregnancy even decreased (60). In conclusion, although women of childbearing age and pregnant women were given varied nutritional or eating advice, there were still grounds for concern regarding the actual quality of their diet. Yu et al. (61) recommended that health practitioners or policymakers should tailor strategies to the quality level of women's diets.

In contrast to dietary patterns, dietary quality assessment can combine large amounts of dietary data into a practical dietary indicator, thereby increasing the feasibility of translating food intake into daily food consumption and providing visualization of the intake of different food groups (62). Studies have shown that stress in women of childbearing age is inversely associated with poor diet quality (63). Borge et al. (64) found a positive association between better maternal diet quality during pregnancy and functioning of the child. A high dietary quality is a strong predictor of chronic diseases (all-cause mortality, cardiovascular disease, and T2DM) (65, 66). However, only a few studies have examined the relationship between diet quality and GDM. This systematic research discovered that high quality diet reduced the risk of developing GDM. As a result, assessment of diet quality can have the potential to be a quick and easy way to screen for dietary habits associated with GDM before or during early pregnancy.

Donazar-Ezcurra et al. (67) found that, compared to pre-pregnancy, healthy dietary measures adopted during pregnancy seem to be ineffective because they require more time to properly curb the development of GDM. However, similar to the results of a previous systematic review (19, 68), we found that high quality diet before and during pregnancy was beneficial for preventing GDM. At present, the well-documented risk factors for GDM include advanced maternal age, family history of diabetes, having a macrosomic baby, non-Caucasian race/ethnicity, being overweight or obese, and cigarette smoking (69). This study found that the impacts of adjustment variables such as family history of diabetes, socioeconomic status, physical activity, and gravidity on the risk of developing GDM should be considered when systematically evaluating outcome analysis.

Some limitations of this meta-analysis should be considered. First, studies were observational, making causal inferences difficult. Although the studies adjusted for some confounders, the possibility of residual confounding cannot be ruled out completely. Most studies accounted for maternal age, BMI, and family history of diabetes; however, most of the included studies did not adjust for GWG, socioeconomic or alcohol status, previous macrosomia, or polycystic ovary syndrome, which may be important risk factors for GDM (70) (Supplementary Figure 1). Secondly, the most studied countries (61%) were the United States and China, where populations have different dietary habits. Although many studies assessed dietary intake with validated measurement tools (e.g., FFQ), these dietary data were self-reported. Additionally, the timing of the dietary data assessment was heterogeneous. We did not know how much time elapsed between the assessment of diet quality and the diagnosis of GDM; in some studies, diet was assessed years before pregnancy, and in others, it was assessed during pregnancy. Finally, no evidence of publication bias based on Egger's test was found in this meta-analysis.

We suggest the following recommendations for future studies. During the sampling and survey phases, studies should be conducted in diverse populations with varying racial/ethnic backgrounds, socioeconomic status, BMI ranges, and diet culture. Research should improve the collection methods of food intake, which can combine contemporary Internet technology such as applets and real-time recording, to capture the complexity of dietary habits more accurately. During the design and analysis phase, appropriate analytical methods should be used, and adjustments for covariates should be demonstrated through causal considerations and graphs, particularly for physical activity, gravidity, and socioeconomic status. Comparability studies should be improved by increasing the uniformity of the timing of dietary assessments and the outcomes measured. When possible, adequately powered randomized controlled trials should be conducted to support better causal inferences. In addition, one should investigate what are the micronutrient levels in women of childbearing age or in pregnant women with high or poor dietary quality.

## 5. Conclusions

In conclusion, positive association between better maternal diet quality during pregnancy and functioning of the child. However, only a few studies have examined the relationship between diet quality and GDM. This study found that higher diet quality (MD, DASH diet, AHEI, PDI, or adherence to national dietary guidelines) before or during pregnancy reduced the prevalence of GDM; while poorer diet quality (higher DII or LCD) increased the risk of developing GDM. And then, the assessment of diet quality can have the potential to be a quick and easy way to screen for dietary habits associated with GDM before or during early pregnancy. Further studies are necessary to ascertain the relationship between food quality and GDM.

## Data availability statement

The original contributions presented in the study are included in the article/Supplementary material, further inquiries can be directed to the corresponding author.

## Author contributions

XG, QZ, and XJ conceived and designed the experiments. XG and QZ performed the experiments and wrote the paper. XG, YP, and YL analyzed the data. XC contributed materials and analysis tools. All authors read and approved the final manuscript prior to submission.
