# Supplementary material for: The effect of diet quality on the risk of developing gestational diabetes mellitus: A systematic review and meta-analysis
Source: Front Public Health. 2023 Jan 9;10:1062304. doi: 10.3389/fpubh.2022.1062304 (PMC9868748; doi:10.3389/fpubh.2022.1062304)
Supplement: Supplementary file 1 [file Table_1.docx]

**Supplemental Table 1**

Supplemental Table 1 Search strategy

| **Database** | **Search strategy** |
| --- | --- |
| **PubMed** | #1 gestational diabetes mellitus[MeSH Terms]  #2 gestational diabetes mellitus[Title/Abstract] OR GDM[Title/Abstract] OR Diabetes, Pregnancy-Induced[Title/Abstract] OR Diabetes, Pregnancy Induced[Title/Abstract] OR Pregnancy-Induced Diabetes[Title/Abstract] OR Gestational Diabetes[Title/Abstract] OR Diabetes Mellitus, Gestational[Title/Abstract] OR Gestational Diabetes Mellitus[Title/Abstract]  #3 #1 OR #2  #4 dietary quality[Title/Abstract] OR dietary quality index[Title/Abstract] OR DQI[Title/Abstract] OR diet score[Title/Abstract] OR diet index[Title/Abstract] OR dietary guidelines[Title/Abstract] OR Recommended Food Score[Title/Abstract] OR priori[Title/Abstract] OR posteriori[Title/Abstract] OR Mediterranean diet scores[Title/Abstract] OR Alternate Mediterranean Diet Score[Title/Abstract] OR AMED[Title/Abstract] OR MDS[Title/Abstract] OR healthy eating index[Title/Abstract] OR diet quality indices[Title/Abstract] OR HEI[Title/Abstract] OR Alternate Healthy Eating Index[Title/Abstract] OR AHEI[Title/Abstract] OR diet balance index[Title/Abstract] OR Dietary diversity scores[Title/Abstract] OR DDS[Title/Abstract] OR Dietary Approaches to Stop Hypertension scores[Title/Abstract] OR DASH[Title/Abstract]  #5 #3 AND #4 |
| **Embase** | #1 'pregnancy diabetes mellitus'/exp  #2 gdm:ti,ab,kw OR 'diabetes, pregnancy-induced':ti,ab,kw OR 'diabetes, pregnancy induced':ti,ab,kw OR 'pregnancy-induced diabetes':ti,ab,kw OR 'gestational diabetes':ti,ab,kw OR 'diabetes mellitus, gestational':ti,ab,kw OR 'gestational diabetes mellitus':ti,ab,kw  #3 #1 OR #2  #4 'dietary quality':ti,ab,kw OR 'dietary quality index':ti,ab,kw OR 'diet score':ti,ab,kw OR 'diet index':ti,ab,kw OR 'dietary guidelines':ti,ab,kw OR 'recommended food score':ti,ab,kw OR priori:ti,ab,kw OR posteriori:ti,ab,kw OR 'mediterranean diet scores':ti,ab,kw OR 'alternate mediterranean diet score':ti,ab,kw OR amed:ti,ab,kw OR mds:ti,ab,kw OR 'healthy eating index':ti,ab,kw OR 'diet quality indices':ti,ab,kw OR 'alternate healthy eating index':ti,ab,kw OR 'diet balance index':ti,ab,kw OR 'dietary diversity scores':ti,ab,kw OR 'dietary approaches to stop hypertension scores':ti,ab,kw OR 'DASH':ti,ab,kw  #5 #3 AND #4 |
| **Cochrane Library** | #1 MeSH descriptor: [Diabetes, Gestational] explode all trees  #2 (gestational diabetes mellitus or GDM or Diabetes, Pregnancy-Induced or Diabetes, Pregnancy Induced or Pregnancy-Induced Diabetes or Gestational Diabetes or Diabetes Mellitus, Gestational or Gestational Diabetes Mellitus):ti,ab,kw  #3 #1 or #2  #4 (dietary quality or dietary quality index or DQI or diet score or diet index or dietary guidelines or Recommended Food Score or priori or posteriori or Mediterranean diet scores or Alternate Mediterranean Diet Score or AMED or MDS or healthy eating index or diet quality indices or HEI or Alternate Healthy Eating Index or AHEI or diet balance index or Dietary diversity scores or DDS or Dietary Approaches to Stop Hypertension scores or DASH):ti,ab,kw  #5 #3 and #4 |
| **Web of Science** | #1 Topic: (gestational diabetes mellitus or GDM or Diabetes, Pregnancy-Induced or Diabetes, Pregnancy Induced or Pregnancy-Induced Diabetes or Gestational Diabetes or Diabetes Mellitus, Gestational or Gestational Diabetes Mellitus) Time span: All years. Index: SCI-EXPANDED, SSCI, A&HCI, CPCI-S, CPCI-SSH, ESCI, CCR-EXPANDED, IC.  #2 Topic: (dietary quality or dietary quality index or DQI or diet score or diet index or dietary guidelines or Recommended Food Score or priori or posteriori or Mediterranean diet scores or Alternate Mediterranean Diet Score or AMED or MDS or healthy eating index or diet quality indices or HEI or Alternate Healthy Eating Index or AHEI or diet balance index or Dietary diversity scores or DDS or Dietary Approaches to Stop Hypertension scores or DASH) Time span: All years. Index: SCI-EXPANDED, SSCI, A&HCI, CPCI-S, CPCI-SSH, ESCI, CCR-EXPANDED, IC.  #3 #1 AND #2 |
| **PsycINFO** | #1 TI gestational diabetes mellitus OR GDM OR Diabetes, Pregnancy-Induced OR Diabetes, Pregnancy Induced OR Pregnancy-Induced Diabetes OR Gestational Diabetes OR Diabetes Mellitus, Gestational OR Gestational Diabetes Mellitus  #2 AB gestational diabetes mellitus OR GDM OR Diabetes, Pregnancy-Induced OR Diabetes, Pregnancy Induced OR Pregnancy-Induced Diabetes OR Gestational Diabetes OR Diabetes Mellitus, Gestational OR Gestational Diabetes Mellitus  #3 #1 OR #2  #4 TI dietary quality OR dietary quality index OR DQI OR diet score OR diet index OR dietary guidelines OR Recommended Food Score OR priori OR posteriori OR Mediterranean diet scores OR Alternate Mediterranean Diet Score OR AMED OR MDS OR healthy eating index OR diet quality indices OR HEI OR Alternate Healthy Eating Index OR AHEI OR diet balance index OR Dietary diversity scores OR DDS OR Dietary Approaches to Stop Hypertension scores OR DASH  #5 AB dietary quality OR dietary quality index OR DQI OR diet score OR diet index OR dietary guidelines OR Recommended Food Score OR priori OR posteriori OR Mediterranean diet scores OR Alternate Mediterranean Diet Score OR AMED OR MDS OR healthy eating index OR diet quality indices OR HEI OR Alternate Healthy Eating Index OR AHEI OR diet balance index OR Dietary diversity scores OR DDS OR Dietary Approaches to Stop Hypertension scores OR DASH  #6 #4 OR #5  #7 #3 AND #6 |
| **CINAHL Complete** | #1 MM gestational diabetes mellitus  #2 TI gestational diabetes mellitus OR GDM OR Diabetes, Pregnancy-Induced OR Diabetes, Pregnancy Induced OR Pregnancy-Induced Diabetes OR Gestational Diabetes OR Diabetes Mellitus, Gestational OR Gestational Diabetes Mellitus  #3 AB gestational diabetes mellitus OR GDM OR Diabetes, Pregnancy-Induced OR Diabetes, Pregnancy Induced OR Pregnancy-Induced Diabetes OR Gestational Diabetes OR Diabetes Mellitus, Gestational OR Gestational Diabetes Mellitus  #4 #1 OR #2 OR #3  #5 TI dietary quality OR dietary quality index OR DQI OR diet score OR diet index OR dietary guidelines OR Recommended Food Score OR priori OR posteriori OR Mediterranean diet scores OR Alternate Mediterranean Diet Score OR AMED OR MDS OR healthy eating index OR diet quality indices OR HEI OR Alternate Healthy Eating Index OR AHEI OR diet balance index OR Dietary diversity scores OR DDS OR Dietary Approaches to Stop Hypertension scores OR DASH  #6 AB dietary quality OR dietary quality index OR DQI OR diet score OR diet index OR dietary guidelines OR Recommended Food Score OR priori OR posteriori OR Mediterranean diet scores OR Alternate Mediterranean Diet Score OR AMED OR MDS OR healthy eating index OR diet quality indices OR HEI OR Alternate Healthy Eating Index OR AHEI OR diet balance index OR Dietary diversity scores OR DDS OR Dietary Approaches to Stop Hypertension scores OR DASH  #7 #5 OR #6  #8 #4 AND #7 |

**Supplemental** **Table 2**

Supplemental Table 2 key findings

| **Authors** | **Dietary assessment tools** | **Key findings** | **OR/RR** |
| --- | --- | --- | --- |
| Tobias, et al. (2012)(30) | semiquantitative FFQ (134 items) + AMED, DASH and AHEI | All 3 scores were inversely associated with GDM risk.  AMED, DASH and AHEI were associated with 24%, 34% and 46% lower risk | RR  AMED: 0.76(0.60,0.95);  DASH: 0.78(0.64, 0.95);  AHEI: 0.75(0.61,0.91) |
| Izadi et al. (2016)(41) | three 24-hour dietary records + MDS, DASH | All 2 scores were inversely associated with GDM risk.  adherence to MD was strongly associated with reduced risk of GDM (80%) compared to DASH (71%). | OR  MDS: 0.22(0.13,0.37);  DASH: 0.29(0.17,0.48) |
| Fulay et al. (2018)(32) | semiquantitative FFQ (140 items) + DASH | DASH was not associated with GDM risk. | OR  1.01(0.96,1.06) |
| Gicevic et al. (2018)(33) | semiquantitative FFQ (131 items) + AHEI | AHEI was inversely associated with GDM risk. | RR  0.63(0.50,0.81) |
| Olmedo-Requena et al. (2019)(45) | FFQ(118 items) + MD index | high MD adherence was associated with GDM risk. | OR  0.61(0.39,0.94) |
| Li et al. (2021)(35) | semiquantitative FFQ (153 items) + Automated Self-Administered 24-hour Dietary Assessment Tool + AMED, DASH, and AHEI | A higher AHEI score reported at 16–22 week was inversely associated with GDM risk.  AMED and DASH was not associated with GDM risk. | RR  AMED:  0.61(0.33,1.15);  DASH:  0.61(0.33,1.15);  AHEI:  0.32(0.16,0.66) |
| Zamani et al. (2019)(43) | 3 day food diaries + overall PDI | The high overall PDI score was inversely associated with GDM risk. | OR  0.47(0.28,0.78) |
| Wang H et al. (2021)(39) | semiquantitative FFQ  (61 items) + overall PDI | PDI score was inversely associated with GDM risk.  the highest quartile of PDI were associated with 57 % reduced odds of GDM compared with women in the lowest quartile of PDI, An interquartile ranges increment in PDI was associated with 29 % decreased odds of GDM. | OR  0.43(0.24,0.77) |
| Chen, Z. L et al. (2021)(34) | semiquantitative FFQ  (131 items) + overall PDI | Overall PDI score was inversely associated with GDM risk. | RR  0.7（0.56,0.87） |
| Tryggvadottir et al. (2021)(46) | FFQ (40 items) + dietary whole-grain (adhered to the national food-based dietary guidelines ) | Whole-grain was inversely associated with GDM risk. | RR  0.50(0.27,0.90) |
| Ding et al. (2021)(37) | semiquantitative FFQ  (61 items) + Compliance with Chinese dietary guidelines for pregnant women | A higher maternal CDGCI-PW score was inversely associated with GDM risk. | OR  0.38(0.31,0.48) |
| Bao et al. (2014)(31) | Semiquantitative FFQ + overall LCD | Overall LCD was positively associated with GDM risk. When LCD scores were modeled as a continuous  variable, we showed 6% higher risk of GDM associated with each 5-unit increment of the overall LCD score. | RR  1.27(1.06,1.51) |
| Looman et al. (2018)(47) | FFQ(101 items) + overall LCD | Overall LCD score, reflecting relatively high fat and protein intake and low carbohydrate intake, was positively associated with GDM risk. | RR  1.54(1.10,2.15) |
| Chen, Q et al. (2021)(36) | quantitative FFQ  (81 items) + overall LCD | overall LCD was not associated with GDM risk ,but it was positively associated with OGTT 1-h glucose. | OR  1.48(0.82,2.69) |
| Dong et al. (2021)(38) | 24-hour dietary recalls for 3 days + overall LCD | overall LCD scores was positively associated with GDM risk. | RR  1.25(1.02,1.53) |
| Shivappa et al. (2019)(42) | semiquantitative FFQ  (147 items) + DII | DII was positively associated with GDM risk. | OR  2.10(1.02,4.34) |
| Zhang, Z et al. (2021)(40) | semiquantitative FFQ  (＞200 items) + DII | DII scores was positively associated with GDM risk.  The association between DII  score and risk of GDM was stronger in pregnant women who were overweight or obese before pregnancy. | OR  1.43(1.05,1.95) |
| Pajunen et al. (2022)(48) | 3 day food diaries + DII | DII scores was positively associated with GDM risk. | OR  1.27(1.08,1.49) |
| Kyozuka H（2022）(44) | FFQ (30 food parameters) + DII | DII was positively associated with GDM risk. | OR  1.75(1.21,2.52) |

Annotate: Alternate Mediterranean diet (AMED), Alternate Healthy Eating Index (AHEI), dietary approaches to stop hypertension (DASH), body mass index (BMI), food-frequency questionnaire (FFQ), score of adherence to the Mediterranean diet (MDS), Mediterranean diet(MD), gestational weight gain(GWG), low-carbohydrate(LCD), plant-based dietindex (PDI), healthful plant-based diet index (hPDI), unhealthful plant-based diet index (uPDI), Chinese Dietary Guidelines Compliance Index for Pregnant Women (CDGCI-PW), dietary inflammatory index (DII).

**Supplemental Table 3**

Supplemental Table 3. Meta-regression of adjustment variables (Group A)

| Meta-regression Number of obs = 16  REML estimate of between-study variance tau2 = 0  % residual variation due to heterogeneity I-squared_res = 16.95%  Proportion of between-study variance explained Adj R-squared = 100.00%  Joint test for all covariates Model F(10,5) = 14.28  With Knapp-Hartung modification Prob > F = 0.0045 | | | | | |
| --- | --- | --- | --- | --- | --- |
| _ES | Coef . | Std . Err . | t | P>t | [95% Conf . Interval] |
| race or ethnicity | 0.0394759 | 0.3172418 | 0.12 | 0.906 | -0.7760202 0.854972 |
| education | -0.2039746 | 0.3852165 | -0.53 | 0.619 | -1.194205 0.78625 |
| socioeconomic status | -0.0285172 | 0.1455194 | -0.20 | 0.852 | -0.4025868 0.3455525 |
| physical activity | -0.6761825 | 0.3453741 | -1.96 | 0.108 | -1.563995 0.2116299 |
| Smoking status | -0.2822325 | 0.4392285 | -0.64 | 0.549 | -1.411305 0.8468403 |
| alcohol status | -0.0449795 | 0.1514337 | -0.30 | 0.778 | -0.4342522 0.3442931 |
| gravidity | 0.8041217 | 0.2861458 | 2.81 | 0.038 | 0.0685606 1.539683 |
| family history of diabetes | 0.0480079 | 0.2807339 | 0.17 | 0.871 | -0.6736417 0.7696575 |
| energy intake | 1.140647 | 0.5574779 | 2.05 | 0.096 | -0.2923951 2.57369 |
| GWG up until the time of the study | 0.6599621 | 0.3234233 | 2.04 | 0.097 | -0.1714238 1.491348 |
| _cons | -2.802755 | 0.7750788 | -3.62 | 0.015 | -4.795158 -0.8103514 |

**Supplemental Table 4**

Supplemental Table 4 Subgroup analysis of adjustment variables in the Group A (including race or ethnicity, BMI, education, socioeconomic status, physical activity, smoking status, alcohol status, gravidity, family history of diabetes, energy intake, GWG up until the time of the study)

| **adjustment variables** | **study** | **OR**  **(95%CI)** | **weight** |
| --- | --- | --- | --- |
| race or ethnicity (no) | Izadi et al. (2016) | 0.29(0.17, 0.48) | 5.65 |
|  | Gicevic et al. (2018) | 0.62(0.49, 0.81) | 7.29 |
|  | Zamani et al.(2019) | 0.47(0.28, 0.78) | 5.69 |
|  | Olmedo-Requena et al.(2019) | 0.61(0.39, 0.94) | 6.16 |
|  | Wang H et al.(2021) | 0.43(0.24, 0.77) | 5.24 |
|  | Izadi et al. (2016) | 0.22(0.13, 0.37) | 5.62 |
|  | Tryggvadottir et al.(2021) | 0.46(0.24, 0.88) | 4.88 |
|  | Ding et al. (2021) | 0.38(0.31, 0.48) | 7.46 |
| Subgroup, DL (I^2^=65.5%，P=0.005) | | 0.42(0.33, 0.54) | 47.95 |
| race or ethnicity (yes) | Tobias, et al. (2012) | 0.77(0.63, 0.95) | 7.52 |
|  | Fulay et al. (2018) | 1.01(0.96, 1.06) | 7.98 |
|  | Chen, Z. L et al.(2021) | 0.69(0.55, 0.87) | 7.40 |
|  | Tobias, et al. (2012) | 0.74(0.60, 0.65) | 7.50 |
|  | Li et al. (2021) | 0.60(0.32, 1.16) | 4.87 |
|  | Li et al. (2021) | 0.31(0.16, 0.65) | 4.54 |
|  | Tobias, et al. (2012) | 0.75(0.59, 0.95) | 7.36 |
|  | Li et al. (2021) | 0.60(0.32, 1.16) | 4.87 |
| Subgroup, DL (I^2^=82.7%，P=0.000) | | 0.73(0.59, 0.89) | 52.05 |
| **Overall, DL (I^2^=91.6%，P=0.000)** | | **0.54(0.43, 0.68)** | **100.0** |
| **adjustment variables** | **study** | **OR**  **(95%CI)** | **weight** |
| BMI (no) | Li et al. (2021) | 0.60(0.32, 1.16) | 4.87 |
|  | Izadi et al. (2016) | 0.29(0.17, 0.48) | 5.65 |
|  | Li et al. (2021) | 0.60(0.32, 1.16) | 4.87 |
|  | Li et al. (2021) | 0.31(0.16, 0.65) | 4.54 |
|  | Izadi et al. (2016) | 0.22(0.13, 0.37) | 5.62 |
| Subgroup, DL (I^2^=55.7%，P=0.060) | | 0.36(0.24, 0.54) | 25.56 |
| BMI (yes) | Wang H et al.(2021) | 0.43(0.24, 0.77) | 5.24 |
|  | Zamani et al.(2019) | 0.47(0.28, 0.78) | 5.69 |
|  | Chen, Z. L et al.(2021) | 0.69(0.55, 0.87) | 7.40 |
|  | Tobias, et al. (2012) | 0.77(0.63, 0.95) | 7.52 |
|  | Fulay et al. (2018) | 1.01(0.96, 1.06) | 7.98 |
|  | Gicevic et al. (2018) | 0.62(0.49, 0.81) | 7.29 |
|  | Tobias, et al. (2012) | 0.74(0.60, 0.65) | 7.50 |
|  | Tobias, et al. (2012) | 0.75(0.59, 0.95) | 7.36 |
|  | Olmedo-Requena et al.(2019) | 0.61(0.39, 0.94) | 6.16 |
|  | Tryggvadottir et al.(2021) | 0.46(0.24, 0.88) | 4.88 |
|  | Ding et al. (2021) | 0.38(0.31, 0.48) | 7.46 |
| Subgroup, DL (I^2^=91.8%，P=0.000) | | 0.63(0.50, 0.79) | 74.44 |
| **Overall, DL (I^2^=91.6%，P=0.000)** | | **0.54(0.24, 0.68)** | **100.00** |
| **adjustment variables** | **study** | **OR**  **(95%CI)** | **weight** |
| education (no) | Wang H et al.(2021) | 0.43(0.24, 0.77) | 5.24 |
|  | Zamani et al.(2019) | 0.47(0.28, 0.78) | 5.69 |
|  | Chen, Z. L et al.(2021) | 0.69(0.55, 0.87) | 7.40 |
|  | Tobias, et al. (2012) | 0.77(0.63, 0.95) | 7.52 |
|  | Izadi et al. (2016) | 0.29(0.17, 0.48) | 5.65 |
|  | Gicevic et al. (2018) | 0.62(0.49, 0.81) | 7.29 |
|  | Tobias, et al. (2012) | 0.74(0.60, 0.65) | 7.50 |
|  | Tobias, et al. (2012) | 0.75(0.59, 0.95) | 7.36 |
|  | Izadi et al. (2016) | 0.22(0.13, 0.37) | 5.62 |
|  | Olmedo-Requena et al.(2019) | 0.61(0.39, 0.94) | 6.16 |
|  | Tryggvadottir et al.(2021) | 0.46(0.24, 0.88) | 4.88 |
| Subgroup, DL (I^2^=72.4%，P=0.000) | | 0.56(0.46, 0.68) | 70.28 |
| education (yes) | Li et al. (2021) | 0.60(0.32, 1.16) | 4.87 |
|  | Fulay et al. (2018) | 1.01(0.96, 1.06) | 7.98 |
|  | Li et al. (2021) | 0.60(0.32, 1.16) | 4.87 |
|  | Li et al. (2021) | 0.31(0.16, 0.65) | 4.54 |
|  | Ding et al. (2021) | 0.38(0.31, 0.48) | 7.46 |
| Subgroup, DL (I^2^=95.4%，P=0.000) | | 0.54(0.30, 0.99) | 29.72 |
| **Overall, DL (I^2^=91.6%，P=0.000)** | | **054(0.43, 0.68)** | **100.00** |
| **adjustment variables** | **study** | **OR**  **(95%CI)** | **weight** |
| socioeconomic status (no) | Zamani et al.(2019) | 0.47(0.28, 0.78) | 5.69 |
|  | Chen, Z. L et al.(2021) | 0.69(0.55, 0.87) | 7.40 |
|  | Tobias, et al. (2012) | 0.77(0.63, 0.95) | 7.52 |
|  | Fulay et al. (2018) | 1.01(0.96, 1.06) | 7.98 |
|  | Li et al. (2021) | 0.60(0.32, 1.16) | 4.87 |
|  | Gicevic et al. (2018) | 0.62(0.49, 0.81) | 7.29 |
|  | Li et al. (2021) | 0.60(0.32, 1.16) | 4.87 |
|  | Tobias, et al. (2012) | 0.75(0.59, 0.95) | 7.36 |
|  | Li et al. (2021) | 0.31(0.16, 0.65) | 4.54 |
|  | Tryggvadottir et al.(2021) | 0.46(0.24, 0.88) | 4.88 |
|  | Tobias, et al. (2012) | 0.74(0.60, 0.65) | 7.50 |
|  | Olmedo-Requena et al.(2019) | 0.61(0.39, 0.94) | 6.16 |
| Subgroup, DL (I^2^=83.3%，P=0.000) | | 0.93(0.89, 0.97) | 94.38 |
| socioeconomic status (yes) | Wang H et al.(2021) | 0.43(0.24, 0.77) | 5.24 |
|  | Izadi et al. (2016) | 0.29(0.17, 0.48) | 5.65 |
|  | Izadi et al. (2016) | 0.22(0.13, 0.37) | 5.62 |
|  | Ding et al. (2021) | 0.38(0.31, 0.48) | 7.46 |
| Subgroup, DL (I^2^=34.1%，P=0.207) | | 0.35(0.29, 0.42) | 5.62 |
| **Overall, DL (I^2^=91.6%，P=0.000)** | | **054(0.43, 0.68)** | **100.00** |
| **adjustment variables** | **study** | **OR**  **(95%CI)** | **weight** |
| physical activity (no) | Zamani et al.(2019) | 0.47(0.28, 0.78) | 5.69 |
|  | Fulay et al. (2018) | 1.01(0.96, 1.06) | 7.98 |
|  | Li et al. (2021) | 0.60(0.32, 1.16) | 4.87 |
|  | Izadi et al. (2016) | 0.29(0.17, 0.48) | 5.65 |
|  | Gicevic et al. (2018) | 0.62(0.49, 0.81) | 7.29 |
|  | Li et al. (2021) | 0.60(0.32, 1.16) | 4.87 |
|  | Izadi et al. (2016) | 0.22(0.13, 0.37) | 5.62 |
|  | Li et al. (2021) | 0.31(0.16, 0.65) | 4.54 |
|  | Olmedo-Requena et al.(2019) | 0.61(0.39, 0.94) | 6.16 |
|  | Tryggvadottir et al.(2021) | 0.46(0.24, 0.88) | 4.88 |
|  | Ding et al. (2021) | 0.38(0.31, 0.48) | 7.46 |
| Subgroup, DL (I^2^=93.8%，P=0.000) | | 0.47(0.33, 0.69) | 64.97 |
| physical activity (yes) | Wang H et al.(2021) | 0.43(0.24, 0.77) | 5.24 |
|  | Chen, Z. L et al.(2021) | 0.69(0.55, 0.87) | 7.40 |
|  | Tobias, et al. (2012) | 0.77(0.63, 0.95) | 7.52 |
|  | Tobias, et al. (2012) | 0.75(0.59, 0.95) | 7.36 |
|  | Tobias, et al. (2012) | 0.74(0.60, 0.65) | 7.50 |
| Subgroup, DL (I^2^=0.0%，P=0.447) | | 0.73(0.65, 0.81) | 35.03 |
| **Overall, DL (I^2^=91.6%，P=0.000)** | | **054(0.43, 0.68)** | **100.00** |
| **adjustment variables** | **study** | **OR**  **(95%CI)** | **weight** |
| smoking status (no) | Li et al. (2021) | 0.60(0.32, 1.16) | 4.87 |
|  | Izadi et al. (2016) | 0.29(0.17, 0.48) | 5.65 |
|  | Gicevic et al. (2018) | 0.62(0.49, 0.81) | 7.29 |
|  | Li et al. (2021) | 0.60(0.32, 1.16) | 4.87 |
|  | Izadi et al. (2016) | 0.22(0.13, 0.37) | 5.62 |
|  | Li et al. (2021) | 0.31(0.16, 0.65) | 4.54 |
|  | Olmedo-Requena et al.(2019) | 0.61(0.39, 0.94) | 6.16 |
|  | Ding et al. (2021) | 0.38(0.31, 0.48) | 7.46 |
| Subgroup, DL (I^2^=69.5%，P=0.002) | | 0.43(0.33, 0.57) | 46.46 |
| smoking status (yes) | Zamani et al.(2019) | 0.47(0.28, 0.78) | 5.69 |
|  | Chen, Z. L et al.(2021) | 0.69(0.55, 0.87) | 7.40 |
|  | Wang H et al.(2021) | 0.43(0.24, 0.77) | 5.24 |
|  | Tobias, et al. (2012) | 0.77(0.63, 0.95) | 7.52 |
|  | Fulay et al. (2018) | 1.01(0.96, 1.06) | 7.98 |
|  | Tobias, et al. (2012) | 0.75(0.59, 0.95) | 7.36 |
|  | Tobias, et al. (2012) | 0.74(0.60, 0.65) | 7.50 |
|  | Tryggvadottir et al.(2021) | 0.46(0.24, 0.88) | 4.88 |
| Subgroup, DL (I^2^=84.7%，P=0.000) | | 0.47(0.57, 0.86) | 53.54 |
| **Overall, DL (I^2^=91.6%，P=0.000)** | | **054(0.43, 0.68)** | **100.00** |
| **adjustment variables** | **study** | **OR**  **(95%CI)** | **weight** |
| alcohol status (no) | Li et al. (2021) | 0.60(0.32, 1.16) | 4.87 |
|  | Izadi et al. (2016) | 0.29(0.17, 0.48) | 5.65 |
|  | Gicevic et al. (2018) | 0.62(0.49, 0.81) | 7.29 |
|  | Li et al. (2021) | 0.60(0.32, 1.16) | 4.87 |
|  | Izadi et al. (2016) | 0.22(0.13, 0.37) | 5.62 |
|  | Li et al. (2021) | 0.31(0.16, 0.65) | 4.54 |
|  | Olmedo-Requena et al.(2019) | 0.61(0.39, 0.94) | 6.16 |
|  | Ding et al. (2021) | 0.38(0.31, 0.48) | 7.46 |
|  | Zamani et al.(2019) | 0.47(0.28, 0.78) | 5.69 |
|  | Tobias, et al. (2012) | 0.77(0.63, 0.95) | 7.52 |
|  | Fulay et al. (2018) | 1.01(0.96, 1.06) | 7.98 |
|  | Tobias, et al. (2012) | 0.75(0.59, 0.95) | 7.36 |
|  | Tobias, et al. (2012) | 0.74(0.60, 0.65) | 7.50 |
|  | Tryggvadottir et al.(2021) | 0.46(0.24, 0.88) | 4.88 |
| Subgroup, DL (I^2^=92.6%，P=0.000) | | 0.54(0.42, 0.69) | 87.35 |
| alcohol status (yes) | Chen, Z. L et al.(2021) | 0.69(0.55, 0.87) | 7.40 |
|  | Wang H et al.(2021) | 0.43(0.24, 0.77) | 5.24 |
| Subgroup, DL (I^2^=54.3%，P=0.139) | | 0.59(0.38, 0.91) | 12.65 |
| **Overall, DL (I^2^=91.6%，P=0.000)** | | **054(0.43, 0.68)** | **100.00** |
| **adjustment variables** | **study** | **OR**  **(95%CI)** | **weight** |
| gravidity (no) | Wang H et al.(2021) | 0.43(0.24, 0.77) | 5.24 |
|  | Zamani et al.(2019) | 0.47(0.28, 0.78) | 5.69 |
|  | Izadi et al. (2016) | 0.29(0.17, 0.48) | 5.65 |
|  | Fulay et al. (2018) | 1.01(0.96, 1.06) | 7.98 |
|  | Izadi et al. (2016) | 0.22(0.13, 0.37) | 5.62 |
|  | Ding et al. (2021) | 0.38(0.31, 0.48) | 7.46 |
| Subgroup, DL (I^2^=96.4%，P=0.000) | | 0.42(0.23, 0.77) | 37.64 |
| gravidity (yes) | Chen, Z. L et al.(2021) | 0.69(0.55, 0.87) | 7.40 |
|  | Li et al. (2021) | 0.60(0.32, 1.16) | 4.87 |
|  | Tobias, et al. (2012) | 0.77(0.63, 0.95) | 7.52 |
|  | Gicevic et al. (2018) | 0.62(0.49, 0.81) | 7.29 |
|  | Li et al. (2021) | 0.60(0.32, 1.16) | 4.87 |
|  | Li et al. (2021) | 0.31(0.16, 0.65) | 4.54 |
|  | Tobias, et al. (2012) | 0.75(0.59, 0.95) | 7.36 |
|  | Tobias, et al. (2012) | 0.74(0.60, 0.65) | 7.50 |
|  | Olmedo-Requena et al.(2019) | 0.61(0.39, 0.94) | 6.16 |
|  | Tryggvadottir et al.(2021) | 0.46(0.24, 0.88) | 4.88 |
| Subgroup, DL (I^2^=8.7%，P=0.362) | | 0.69(0.62, 0.76) | 62.36 |
| **Overall, DL (I^2^=91.6%，P=0.000)** | | **054(0.43, 0.68)** | **100.00** |
| **adjustment variables** | **study** | **OR**  **(95%CI)** | **weight** |
| family history of diabetes  (no) | Zamani et al.(2019) | 0.47(0.28, 0.78) | 5.69 |
|  | Li et al. (2021) | 0.60(0.32, 1.16) | 4.87 |
|  | Fulay et al. (2018) | 1.01(0.96, 1.06) | 7.98 |
|  | Izadi et al. (2016) | 0.29(0.17, 0.48) | 5.65 |
|  | Li et al. (2021) | 0.60(0.32, 1.16) | 4.87 |
|  | Li et al. (2021) | 0.31(0.16, 0.65) | 4.54 |
|  | Izadi et al. (2016) | 0.22(0.13, 0.37) | 5.62 |
|  | Gicevic et al. (2018) | 0.62(0.49, 0.81) | 7.29 |
|  | Ding et al. (2021) | 0.38(0.31, 0.48) | 7.46 |
| Subgroup, DL (I^2^=94.8%，P=0.000) | | 0.46(0.30, 0.71) | 53.97 |
| family history of diabetes  (yes) | Chen, Z. L et al.(2021) | 0.69(0.55, 0.87) | 7.40 |
|  | Wang H et al.(2021) | 0.43(0.24, 0.77) | 5.24 |
|  | Tobias, et al. (2012) | 0.77(0.63, 0.95) | 7.52 |
|  | Olmedo-Requena et al.(2019) | 0.61(0.39, 0.94) | 6.16 |
|  | Tryggvadottir et al.(2021) | 0.46(0.24, 0.88) | 4.88 |
|  | Tobias, et al. (2012) | 0.75(0.59, 0.95) | 7.36 |
|  | Tobias, et al. (2012) | 0.74(0.60, 0.65) | 7.50 |
| Subgroup, DL (I^2^=0.5%，P=0.420) | | 0.71(0.64, 0.79) | 46.07 |
| **Overall, DL (I^2^=91.6%，P=0.000)** | | **054(0.43, 0.68)** | **100.00** |
| **adjustment variables** | **study** | **OR**  **(95%CI)** | **weight** |
| energy intake (no) | Li et al. (2021) | 0.60(0.32, 1.16) | 4.87 |
|  | Gicevic et al. (2018) | 0.62(0.49, 0.81) | 7.29 |
|  | Izadi et al. (2016) | 0.29(0.17, 0.48) | 5.65 |
|  | Li et al. (2021) | 0.60(0.32, 1.16) | 4.87 |
|  | Li et al. (2021) | 0.31(0.16, 0.65) | 4.54 |
|  | Izadi et al. (2016) | 0.22(0.13, 0.37) | 5.62 |
|  | Olmedo-Requena et al.(2019) | 0.61(0.39, 0.94) | 6.16 |
|  | Tryggvadottir et al.(2021) | 0.46(0.24, 0.88) | 4.88 |
|  | Ding et al. (2021) | 0.38(0.31, 0.48) | 7.46 |
| Subgroup, DL (I^2^=65.1%，P=0.003) | | 0.43(0.34, 0.56) | 51.30 |
| energy intake (yes) | Chen, Z. L et al.(2021) | 0.69(0.55, 0.87) | 7.40 |
|  | Wang H et al.(2021) | 0.43(0.24, 0.77) | 5.24 |
|  | Zamani et al.(2019) | 0.47(0.28, 0.78) | 5.69 |
|  | Tobias, et al. (2012) | 0.77(0.63, 0.95) | 7.52 |
|  | Fulay et al. (2018) | 1.01(0.96, 1.06) | 7.98 |
|  | Tobias, et al. (2012) | 0.75(0.59, 0.95) | 7.36 |
|  | Tobias, et al. (2012) | 0.74(0.60, 0.65) | 7.50 |
| Subgroup, DL (I^2^=85.4%，P=0.000) | | 0.72(0.52, 0.89) | 48.70 |
| **Overall, DL (I^2^=91.6%，P=0.000)** | | **054(0.43, 0.68)** | **100.00** |
| **adjustment variables** | **study** | **OR**  **(95%CI)** | **weight** |
| GWG up until the time of the study (no) | Chen, Z. L et al.(2021) | 0.69(0.55, 0.87) | 7.40 |
|  | Wang H et al.(2021) | 0.43(0.24, 0.77) | 5.24 |
|  | Zamani et al.(2019) | 0.47(0.28, 0.78) | 5.69 |
|  | Tobias, et al. (2012) | 0.77(0.63, 0.95) | 7.52 |
|  | Tobias, et al. (2012) | 0.75(0.59, 0.95) | 7.36 |
|  | Tobias, et al. (2012) | 0.74(0.60, 0.65) | 7.50 |
|  | Li et al. (2021) | 0.60(0.32, 1.16) | 4.87 |
|  | Gicevic et al. (2018) | 0.62(0.49, 0.81) | 7.29 |
|  | Izadi et al. (2016) | 0.29(0.17, 0.48) | 5.65 |
|  | Li et al. (2021) | 0.60(0.32, 1.16) | 4.87 |
|  | Li et al. (2021) | 0.31(0.16, 0.65) | 4.54 |
|  | Izadi et al. (2016) | 0.22(0.13, 0.37) | 5.62 |
|  | Olmedo-Requena et al.(2019) | 0.61(0.39, 0.94) | 6.16 |
|  | Tryggvadottir et al.(2021) | 0.46(0.24, 0.88) | 4.88 |
| Subgroup, DL (I^2^=67.9%，P=0.000) | | 0.55(0.46, 0.66) | 84.57 |
| GWG up until the time of the study (yes) | Fulay et al. (2018) | 1.01(0.96, 1.06) | 7.98 |
|  | Ding et al. (2021) | 0.38(0.31, 0.48) | 7.46 |
| Subgroup, DL (I^2^=98.6%，P=0.000) | | 0.62(0.24, 1.62) | 15.43 |
| **Overall, DL (I^2^=91.6%，P=0.000)** | | **054(0.43, 0.68)** | **100.00** |
